# A non-genetic engineering platform for rapidly generating and expanding cancer-specific armed T cells

**DOI:** 10.1186/s12929-023-00929-z

**Published:** 2023-05-31

**Authors:** Yi-Jou Chen, Michael Chen, Tian-Lu Cheng, Yi-Shan Tsai, Chang-Hung Wang, Che-Yi Chen, Tung-Yun Wu, Shey-Cherng Tzou, Kai-Hung Wang, Jing-Jy Cheng, An-Pei Kao, Shyr-Yi Lin, Kuo-Hsiang Chuang

**Affiliations:** 1 Graduate Institute of Pharmacognosy, Taipei Medical University, 250 Wu-Hsing Street, Taipei, Taiwan; 2 Drug Development and Value Creation Research Center, Kaohsiung Medical University, Kaohsiung, Taiwan; 3 Graduate Institute of Medicine, College of Medicine, Kaohsiung Medical University, Kaohsiung, Taiwan; 4 Department of Biomedical Science and Environmental Biology, Kaohsiung Medical University, Kaohsiung, Taiwan; 5 Master Program in Clinical Genomics and Proteomics, Taipei Medical University, Taipei, Taiwan; 6 Ph.D. Program in Clinical Drug Development of Herbal Medicine, Taipei Medical University, Taipei, Taiwan; 7 Departmet of Biological Science and Technology, National Yang Ming Chiao Tung University, Hsinchu, Taiwan; 8 Center for Reproductive Medicine, Kuo General Hospital, Tainan, Taiwan; 9 National Research Institute of Chinese Medicine, Ministry of Health and Welfare, Taipei, Taiwan; 10 CytoArm Co., Ltd, Taipei, Taiwan; 11 Division of Gastroenterology and Hepatology, Department of Internal Medicine, Taipei Medical University Hospital, 252 Wu-Hsing Street, Taipei, Taiwan; 12 Department of General Medicine, School of Medicine, College of Medicine, Taipei Medical University, Taipei, Taiwan; 13 TMU Research Center of Cancer Translational Medicine, Taipei Medical University, Taipei, Taiwan; 14 Traditional Herbal Medicine Research Center of Taipei Medical University Hospital, Taipei, Taiwan; 15 Ph.D Program in Biotechnology Research and Development, Taipei Medical University, Taipei, Taiwan; 16 The Ph.D. Program of Translational Medicine, Taipei Medical University, Taipei, Taiwan

**Keywords:** Adoptive T cell therapy, Cancer-specific T cell, Bispecific antibody (BsAb), Virus-free engineering platform, BsAb-armed T cell

## Abstract

**Background:**

Cancer-specific adoptive T cell therapy has achieved successful milestones in multiple clinical treatments. However, the commercial production of cancer-specific T cells is often hampered by laborious cell culture procedures, the concern of retrovirus-based gene transfection, or insufficient T cell purity.

**Methods:**

In this study, we developed a non-genetic engineering technology for rapidly manufacturing a large amount of cancer-specific T cells by utilizing a unique anti-cancer/anti-CD3 bispecific antibody (BsAb) to directly culture human peripheral blood mononuclear cells (PBMCs). The anti-CD3 moiety of the BsAb bound to the T cell surface and stimulated the differentiation and proliferation of T cells in PBMCs. The anti-cancer moiety of the BsAb provided these BsAb-armed T cells with the cancer-targeting ability, which transformed the naïve T cells into cancer-specific BsAb-armed T cells.

**Results:**

With this technology, a large amount of cancer-specific BsAb-armed T cells can be rapidly generated with a purity of over 90% in 7 days. These BsAb-armed T cells efficiently accumulated at the tumor site both in vitro and in vivo*.* Cytotoxins (perforin and granzyme) and cytokines (TNF-α and IFN-γ) were dramatically released from the BsAb-armed T cells after engaging cancer cells, resulting in a remarkable anti-cancer efficacy. Notably, the BsAb-armed T cells did not cause obvious cytokine release syndrome or tissue toxicity in SCID mice bearing human tumors.

**Conclusions:**

Collectively, the BsAb-armed T cell technology represents a simple, time-saving, and highly safe method to generate highly pure cancer-specific effector T cells, thereby providing an affordable T cell immunotherapy to patients.

**Supplementary Information:**

The online version contains supplementary material available at 10.1186/s12929-023-00929-z.

## Background

Adoptive T cell immunotherapies have demonstrated great anti-cancer effects in several clinical trials and have emerged as a new focus for therapeutic cancer strategies in recent years [1–3]. During the adoptive T cell immunotherapy, patients’ peripheral blood mononuclear cells (PBMCs) are isolated from surrounding blood and incubated with an anti-CD3 antibody (clone OKT3) and interleukin 2 (IL-2) to promote their proliferation and differentiation into effector T cells, which are then injected back into the patients for cancer treatment [4, 5]. The binding of specific T cell receptors (TCR) to major histocompatibility complex (MHC)/antigen complexes on cancer cells induces the TCR/CD3-mediated signal transduction pathway and stimulates the release of specific toxins (perforin and granzyme) for cancer elimination [6, 7]. However, most effector T cells generated using these established cultivation methods lack cancer specificity [8]. Isolated autologous tumor-infiltrating T cells have been shown remarkable effects in treating several types of tumors in clinical trials; however, the process of isolating and expanding tumor-infiltrating T cells from patients is technically challenging due to the relatively low amount of effector T cells that can be isolated from tumor regions [9, 10]. Moreover, some clinical trial reports have indicated that malignant tumors tend to develop immune escape mechanisms (such as reducing MHC expression), which can seriously interfere with the ability of T cells to recognize and kill tumors [11–13].

Chimeric antigen receptor (CAR)-T cell technology is the most well-known cellular immunotherapy due to its substantial success in treating hematological tumors and has been approved by the US Food and Drug Administration (FDA) and the European Medicines Agency (EMA) [14–16]. The CAR-T cell manufacturing platform is based on the ex vivo genetic engineering of a patient’s T cells to express CAR, followed by the reinfusion of the T cells into the patient. CAR comprises an extracellular, cancer-specific antibody fused with the intracellular signaling activation domains of CD3 and co-activation ligands, such as CD28 or 4-1BB [17, 18], which provide engineered T cells with an MHC‐independent mechanism through which to kill cancer cells directly. However, the gene transfer technology used in current CAR T cells primarily depends on retrovirus or lentivirus systems, which will randomly insert the CAR gene into the T cell genome and may pose a potential carcinogenic risk [19, 20]. To avoid this risk, the FDA limits the infection ratio between viral particles and T cells, resulting in batch-to-batch variations and sometimes unsatisfactory CAR expression on CAR-T cells [21, 22]. Moreover, a clinical case reports examining treatment with CD19 CAR-T cells indicated that the CAR gene was unintentionally introduced into CD19-positive leukemic B-cells during the CAR-T cell manufacturing process, resulting in a highly malignant, CD19-negative B-cell leukemia in the patient [23]. Therefore, a non-genetic engineering-based method for constructing cancer-specific T cells has become a focus of current cancer research.

**Fig. 1:**
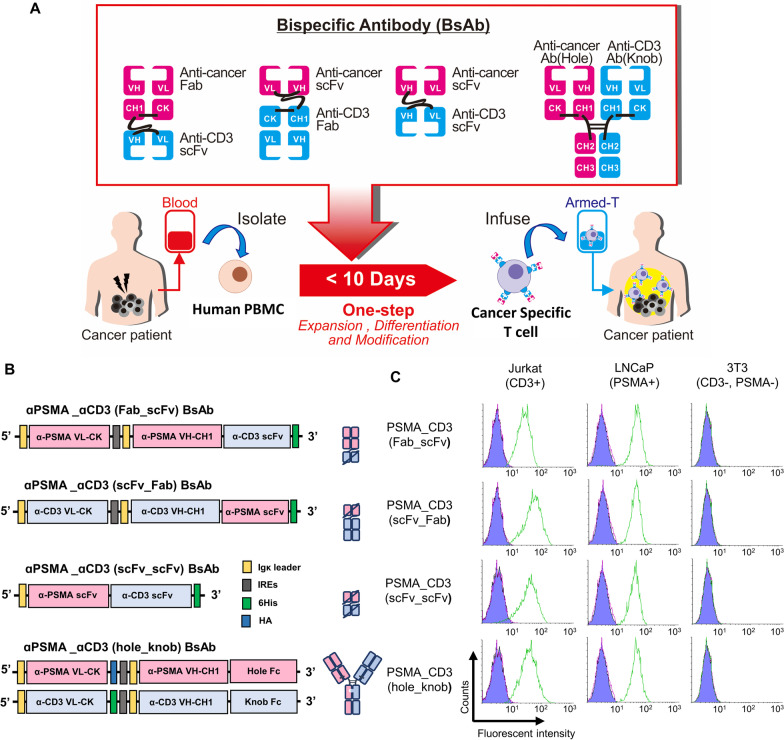
A bispecific antibody (BsAb)-based culturing platform for one-step producing the prostate cancer-specific T cells. **A** Schematic of BsAb-based culturing platform, culture the human PBMCs with anti-PSMA/anti-CD3 BsAb and stimulated with IL-2, which can induce PBMCs proliferation and differentiation into CD3^+^ CD8^+^ T cells. During this cultivation process, the BsAb anti-CD3 end can bind to CD3^+^ T cell surface and cause the BsAb-armed T cells production. Moreover, the BsAb anti-cancer end allows BsAb-armed T cell to target cancer cells and activate CD3 downstream signaling to kill cancer cells specifically. **B** Four kinds of bispecific antibody structures were developed, namely anti-PSMA/anti-CD3 (Fab-scFv), anti-PSMA/anti-CD3(scFv-Fab), anti-PSMA/anti-CD3(scFv-scFv), and anti-PSMA/anti-CD3 (hole-knob). IgK leader, IgK leader sequence. IRES, internal ribosome entry site. 6His, six-histidine tag. Knob Fc, a human IgG1 heavy chain with knob mutations (S354C, T366W). Hole Fc, a human IgG1 heavy chain with hole mutations (D399K, E356K). **C** The function of each BsAb to recognize Jurkat, LNCaP, or 3T3. The results were analyzed by flow cytometry

In this study, we present a non-viral method for the generation of cancer-specific T cells (named bispecific antibody [BsAb]-armed T cells) using a novel anti-cancer/anti-CD3 BsAb to mediate the one-step culturing, expansion, and arming of effector T cells derived from human PBMCs (Fig. 1A). There are four structures of anti-cancer/anti-CD3 BsAbs with different CD3 binding affinities: Fab-scFv, scFv-Fab, scFv-scFv, and antibody (knob-into-hole) structures, were produced by a mammalian cell expression system (Fig. 1B). We compared the effects of these four types of BsAb on T cell differentiation and expansion from PBMCs and assessed the level of BsAb found on the T cell surface. In addition, we examined the efficacy of these BsAb-armed T cells on tumor targeting, biodistribution, cytokine and cytotoxin release, body toxicity, and tumor-killing using in vitro and in vivo prostate tumor models. This new BsAb-armed T cell technology presents a simple, time-saving, and highly safe method for generating highly pure, cancer-specific, and effector T cells, which can provid a better T cell immunotherapy to patients.

## Methods

### Cell culture

LNCaP, PC-3, and Jurkat cells (American Type Culture Collection, Manassas, VA, U.S.A.) were maintained in a culture medium containing RPMI-1640, 10% FBS, and 100 units/mL of penicillin and streptomycin (Invitrogen, Carlsbad, CA, U.S.A.). NIH/3T3 cells (American Type Culture Collection, Manassas, VA, U.S.A.) were maintained in a culture medium containing DMEM, 10% FBS, and 100 units/mL of penicillin and streptomycin (Invitrogen, Carlsbad, CA, U.S.A.). Expi293 cells were maintained in Expi293 Expression Medium (Invitrogen, Carlsbad, CA, U.S.A.) with 8% CO_2_ and on an orbital shaker (120 rpm) at 37 °C.

### Construction of anti-cancer/anti-CD3 BsAbs

To construct anti-cancer/anti-CD3 BsAbs, anti-prostate-specific membrane antigen (PSMA) antibody (the sequence is derived from the patented antibody J591) was fused with anti-CD3 antibody (the sequence is derived from the clinical antibody BiTE^®^) and then constructed into anti-PSMA/anti-CD3 BsAbs by using gene synthesis (Genescript Corp. Piscataway, NJ). Four different structures of BsAb were developed, namely anti-PSMA Fab/anti-CD3 scFv, anti-PSMA scFv/anti-CD3 Fab, anti-PSMA scFv/anti-CD3 scFv, and anti-PSMA antibody (with hole Fc)/anti-CD3 antibody (with knob Fc). The BsAb sequences were digested with StuI and ClaI and then subcloned into pLNCX vector (Clontech, Mountain View, CA, USA) containing a six-histidine (His) or hemagglutinin (HA) epitope to form the following plasmid DNA: pLNCX-anti-PSMA Fab/anti-CD3 scFv BsAb-His, pLNCX-anti-PSMA scFv/anti-CD3 Fab BsAb-His, pLNCX-anti-PSMA scFv/anti-CD3 scFv BsAb-His, and pLNCX-anti-PSMA antibody (with hole Fc, His)/ pLNCX-anti-CD3 antibody (with konb Fc, HA).

### Anti-cancer/anti-CD3 BsAbs production and purification

To produce anti-cancer/anti-CD3 BsAbs, the BsAb plasmids (30 μg) were transfected into Expi293 cells with ExpiFectamine (Thermo Fisher Scientific, Waltham, MA, USA). The BsAbs were harvested 5 days after the plasmid transfection. Pure anti-PSMA Fab/anti-CD3 scFv, anti-PSMA scFv/anti-CD3 Fab, and anti-PSMA scFv/anti-CD3 scFv were obtained by using HiTrap columns (GE Healthcare, Munich, Germany). Pure anti-PSMA hole /anti-CD3 konb were obtained by using HiTrap and HA columns (GE Healthcare). The concentrations of purified BsAbs were determined by Pierce™ BCA protein assay kit (Thermo Fisher Scientific). The purity levels of BsAbs were analyzed by non-reducing or reducing sodium dodecyl sulfate polyacrylamide gel electrophoresis (SDS-PAGE).

### Analysis of cancer or CD3 recognition ability of anti-cancer/anti-CD3 BsAbs by flow cytometry

To analyze the cancer or CD3 recognition ability of BsAbs, the LNCaP (PSMA^+^), or Jurkat (CD3^+^) cells were suspended at 3 × 10^5^ cells/tube in PBS buffer containing 0.05% (w/v) BSA and incubated with anti-PSMA/anti-CD3 BsAbs (1 μg/mL). The cell surface BsAb were detected by anti-His monoclonal IgG antibody (1 μg/mL, clone MCA1396G, AbD Serotec, Kidlington, Oxford, UK) and FITC-conjugated goat anti-mouse IgG Fc antibody (Jackson ImmunoResearch, 1 μg/mL). Surface fluorescence of the viable cells was measured using a flow cytometer (BD Biosciences, San Jose, CA, U.S.A.), and fluorescence intensities were analyzed with Flowing Software 2 (University of Turku, Turku, Finland).

### The preparation of anti-cancer BsAb-armed T cells

Human PBMCs were separated from the whole blood of healthy donors by Ficoll density gradient centrifugation (GE Healthcare) and washed with PBS. The PBMCs were seeded and cultured to a concentration of 2 × 10^6^ cells in AIM-V medium (Gibco, MA, USA) containing 10–100 ng/mL of functional grade OKT3 (Thermo Fisher Scientific) or BsAbs and 3000 IU/mL human recombinant IL-2 (PeproTech Inc., USA) with 5% CO_2_ at 37 °C. The anti-cancer BsAb-armed T cells were harvested after 7–10 days of ex vivo expansion. The phenotypes of T cells and anti-cancer BsAb-armed T cells were detected by alexa fluor 488-conjugated CD3 monoclonal antibody (OKT3), FITC-conjugated CD4 monoclonal antibody (SK3), FITC-conjugated CD8a monoclonal antibody (OKT8), APC-eFluor 780-conjugated CD25 monoclonal antibody (BC96), and PE-conjugated CD279 (PD-1) antibody using a flow cytometer (BD Biosciences). The BsAbs arming levels on anti-cancer BsAb-armed T cells surfaces were detected by mouse anti-His monoclonal IgG antibody and FITC-conjugated goat anti-mouse IgG Fc antibody. The OKT3 retention levels on T cell surfaces were detected by FITC-conjugated goat anti-mouse IgG Fc antibody. Blood collection for research was approved by Taipei Medical University’s Institutional Review Board (TMU-JIRB N201606001).

### T cells armed with different BsAb concentrations

The T cells were incubated with 200, 40, 8, 1.6, or 0.32 nM BsAbs for 1 h at 37 °C. After removing unbound antibodies with extensive PBS washing, the T cells’ surface BsAb level was analyzed by staining with a phycoerythrin (PE)-conjugated goat anti-human IgG Fab antibody. The surface fluorescence of viable cells was measured using a flow cytometer (BD Biosciences), and fluorescence intensities were analyzed with the Flowing Software 2 (University of Turku, Turku, Finland).

### Ex vivo autologous activated assay of BsAb-armed T cells

The T cells or BsAb-armed T cells were incubated with 43, 14, 4.7, 1.6, 0.5, 0.2, 0.06, or 0.02 nM OKT3 or BsAbs for 1 h at 37 °C. After the removal of unbound antibodies by extensive washing with AIM-V medium, the T cells and BsAb-armed T cells were incubated for 16 h at 37 °C. The supernatants were harvested and tested by ELISA for human IL-2, IL-6, TNF-α, and IFN-γ (Thermo Fisher Scientific).

### Cancer killing efficiency of BsAb-armed T cells

LNCaP cells and PC-3 cells were seeded at the density of 10^4^ cells/well in 96-well plates and incubated at 37 °C. T cells or BsAb-armed T cells were added at effector-to-target (E:T) ratios of 3:1, 5:1, or 10:1 and incubated for 16 h at 37 °C. The supernatants were harvested and tested by CytoTox 96 Non-Radioactive Cytotoxicity Assay (Promega, Madison, WI, USA). The killing efficacy of T cells or BsAb-armed T cells against target cells was assessed by calculating absorbance values with the following equation: Cytotoxicity (%) = (Experimental − Effector spontaneous − Target spontaneous) / (Target maximum − Target spontaneous) × 100%. For cytokine assays, the supernatants harvested from the cytotoxicity assay were tested by ELISA for human IL-2, TNF-α, IFN-γ (Thermo Fisher Scientific), Perforin (Mabtech, Sweden), and Granzyme B (R&D Systems, Minneapolis, MN, USA).

### Time-lapse live video microscopy

Cancer cells were seeded at the density of 2 × 10^4^ cells/well in 96-well plates and incubated at 37 °C. After 24 h, the T cells or BsAb-armed T cells were added at E:T ratios of 2:1 at 37 °C. The images were recorded in intervals of 5 min for 10 h using a Cytation 3 multi-mode reader (Bio-Tek, Winooski, VT, USA). Image analysis was performed using Movie Maker software.

### In vivo BsAbs retention time on BsAb-armed T cell surfaces

SCID mice were injected intravenously with 10^7^ T cells or BsAb-armed T cells and then sacrificed after 24, 48, 72, or 96 h. The lymphocytes and splenocytes were collected and incubated with FITC-conjugated CD8a monoclonal antibody and phycoerythrin (PE)-conjugated goat anti-human IgG Fab antibody. The surface fluorescence was measured by a flow cytometer (Sony SA3800 Spectral Analyzer), and fluorescence intensities were analyzed using the SA3800 software program (Sony Biotechnology Inc, San Jose, CA).

### Pharmacokinetic property assessment of BsAbs in mice models

C57BL/6 mice were injected intravenously with BsAbs or human IgG antibody at 5 mg/kg. Mice were bled prior to dosing and at 5 and 30 min; 1, 3, and 12 h; and on days 1, 2, 3, and 4 after injection. Serum samples were detected by anti-human IgG Fab antibody. Signals were developed by using ABTS substrate, and the absorbance at 405 nm was measured by ELISA reader (Epoch™, BioTek, USA). Analysis of pharmacokinetic data was performed with WinNonlin software (version 3, Pharsight, Mountain View, CA). The serum albumin, alanine transaminase (ALT), and aspartate transaminase (AST) levels were determined in the undiluted sample as commissioned by the Laboratory Animal Center of Taipei Medical University with the Vet Test analyzer.

### In vivo cytokine release assays

SCID mice were injected intravenously with 10^7^ T cells or BsAb-armed T cells. Then, mice bearing T cells were injected with or without OKT3 at 0.5 mg/kg. Mice were bled prior to dosing and at 24, 48, and 72 h after injection. Serum samples were prepared and frozen at − 80 °C until used. The human cytokines were tested by ELISA for human IL-2, IL-6, TNF-α, and IFN-γ (Thermo-Fisher Scientific). Rectal temperatures of the mice were measured before each time-point bleeding by inserting a rectal thermocouple probe (Bio-Cando biotechnology Inc., Taipei, Taiwan) until a stable reading was obtained.

### T cell or BsAb-armed T cell conjugation with NIR-797

T cells or BsAb-armed T cells were washed with PBS and incubated with 100 μM NIR-797-isothiocyanate (Sigma-Aldrich, St Louis, MO, USA) for 1 h at 4 °C. After using the AIM-V medium to stop the reaction, the T cells or BsAb-armed T cells were washed twice with PBS and resuspended in the AIM-V medium for use.

### The bio-distribution of BsAb-armed T cells

SCID mice were implanted with LNCaP cells (5 × 10^6^ cells/mouse). After tumor growth to 100 mm^3^, mice were injected intravenously with 10^7^ cells/mouse T cells or BsAb-armed T cells which were modified with NIR-797 (Sigma-Aldrich). The distributions and accumulations of fluorescent signals were obtained 24 h post-injection by IVIS Lumina XRMS (PerkinElmer Inc, Waltham, MA, USA) equipped with a Cy5.5 filter (excitation 660 nm, emission 710 nm). The image data were analyzed with Living Image software version 2.50 (Caliper Life Sciences, Hopkinton, MA, USA).

### In vivo tumor growth inhibition of BsAb-armed T cells

SCID mice were implanted with LNCaP cells (5 × 10^6^ cells/mouse). After tumor growth to 50 mm^3^, mice were injected intravenously with T cells (3 × 10^6^ cells/mouse), BsAb-armed T cells (3 × 10^6^ cells/mouse), BsAbs (0.3 mg/kg), or medium twice per week for 2 weeks. The body weights were recorded and tumor volumes were calculated by using the volume formula: V = (length × width × height)/2. Mice were sacrificed after the treatment ended, and the tumors were collected for photography and weighing.

### Statistical analyses

**Fig. 2:**
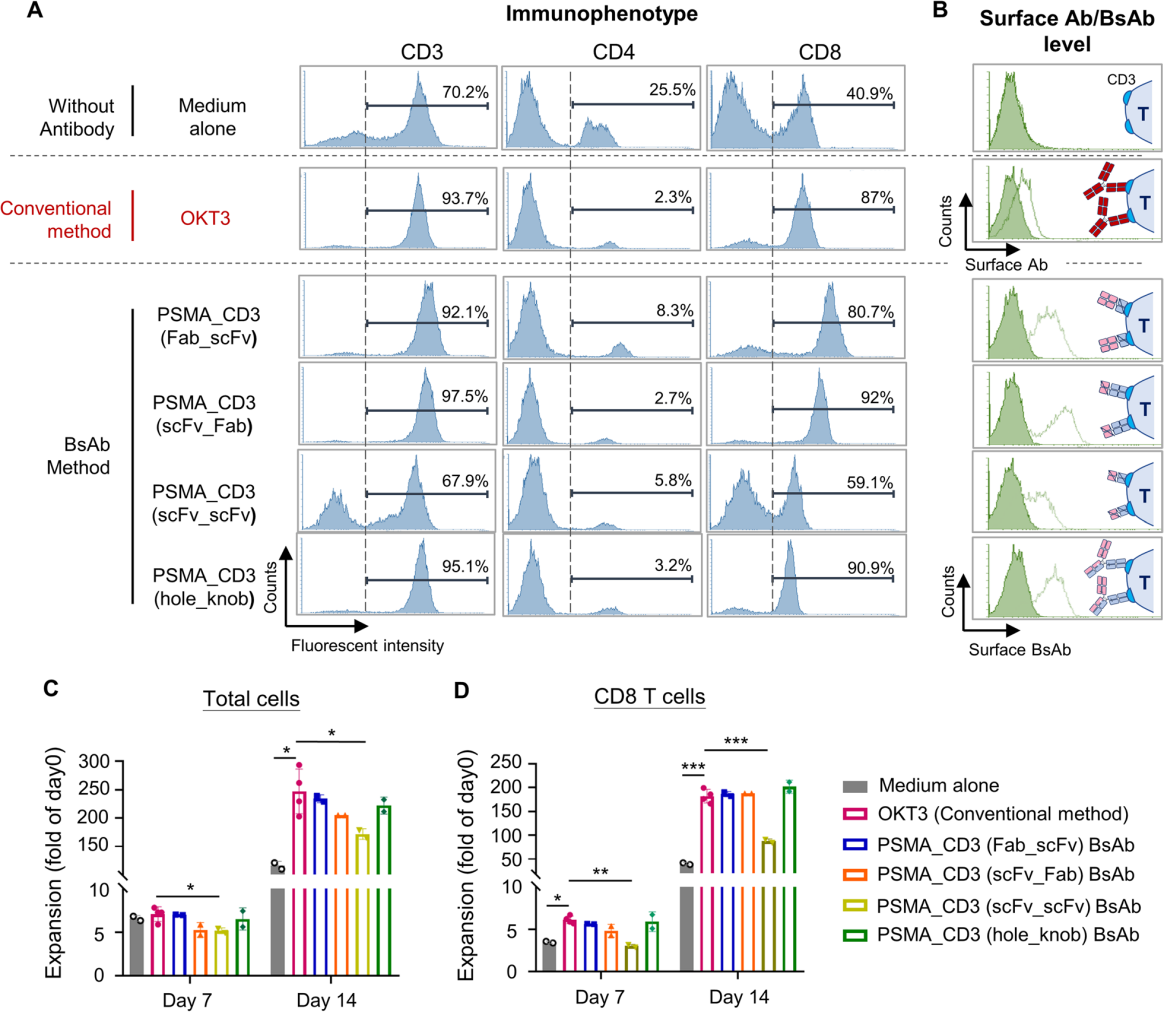
The demonstration of BsAb-based culturing platform. Human PBMCs were isolated from the blood of healthy donors and co-cultured with four types BsAb or traditional mouse anti-CD3 antibody (OKT3), respectively. After 14 day culturing, the (**A**) T cell population was analyzed by using flow cytometry with anti-CD3 antibody, anti-CD4 antibody, or anti-CD8 antibody staining. **B** The surface OKT3 or BsAb were detected by anti-mouse antibody or anti-His antibody respectively. The cell expansion rate of (**C**) total cells or (**D**) CD3^+^ CD8^+^ T cells cultured by OKT3 or BsAbs were calculated on consecutive days. Key: bar, SD; *p < 0.05; **p < 0.01; ***p < 0.001

**Fig. 3:**
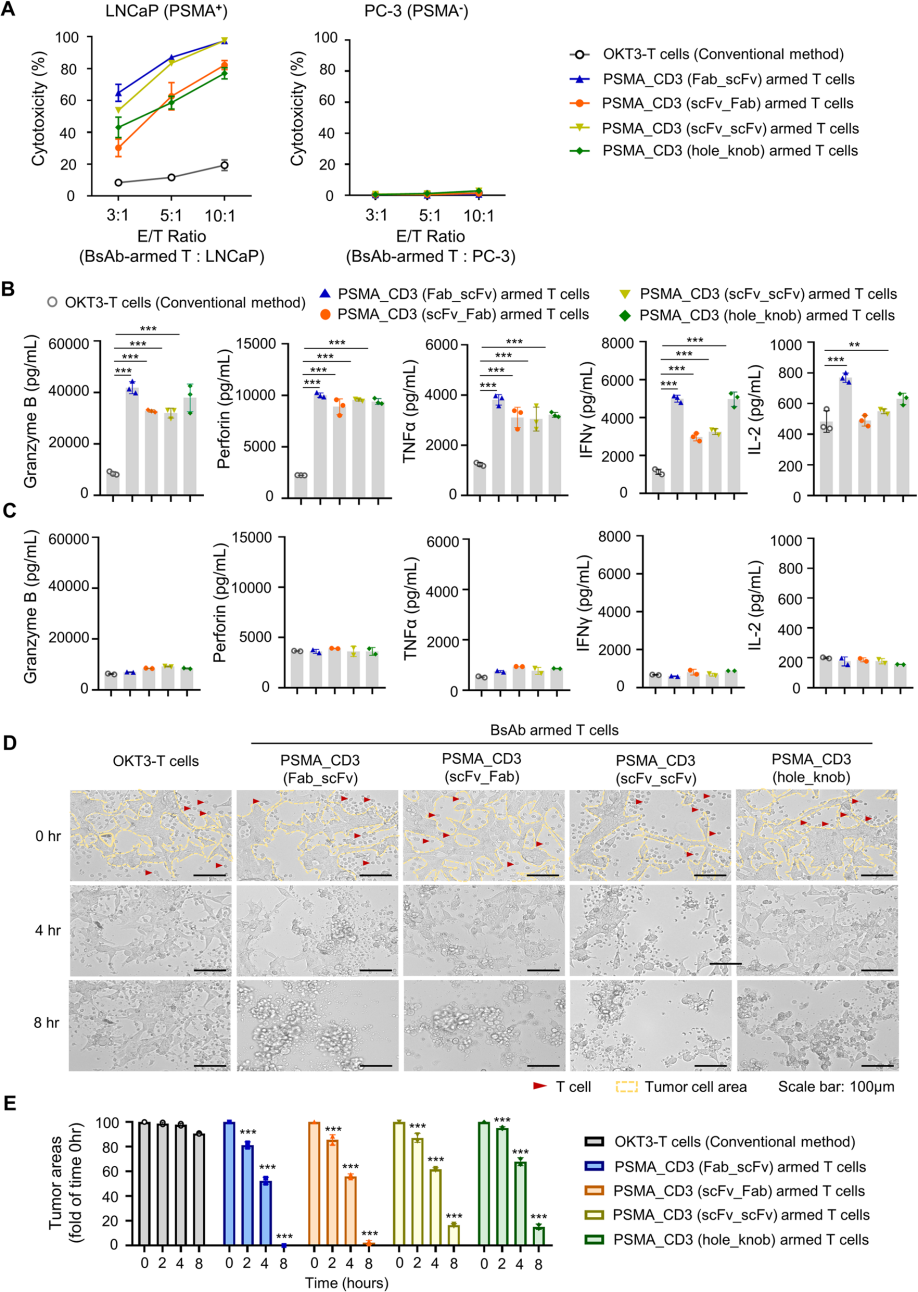
BsAb-armed T cells enhanced the cancer-killing capacity. T cells cultured with anti-PSMA/anti-CD3 BsAbs (called BsAb-armed T cell) or OKT3 (called OKT3-T cell) were applied at different ratios (effector cell/target cell ratios of 3:1, 5:1, or 10:1) and added to **A** LNCaP or PC-3 for 18 h. The anti-cancer efficiency of each group was detected by using the CytoTox 96 Non-Radioactive Cytotoxicity Assay. **B, C** The cytotoxins (perforin and granzyme B) and cytokines (IL-2, INF-γ, and TNF-α) released from T cell co-cultured with LNCaP or PC-3 cells were analyzed by commercial ELISA kits. **D** The ex vivo T cell targeting and killing efficiency to LNCaP were observed by real-time and live-cell imaging system. **E** The tumor cell area were be qualified by ImageJ and compared with control in each timepoint. Bar, SD; **p < 0.01; ***p < 0.001

**Fig. 4:**
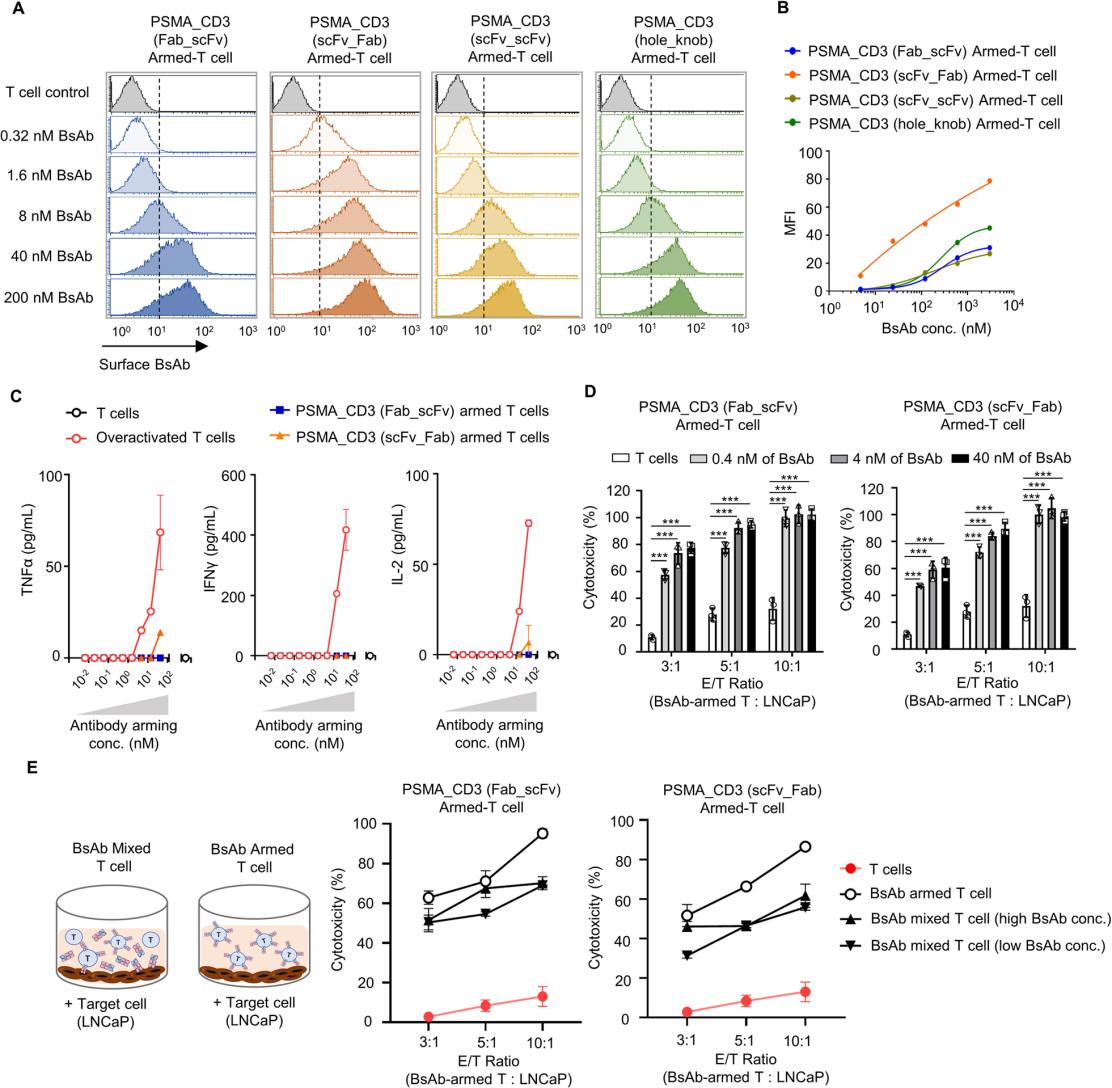
BsAb-armed T cells could be easily adjusted the cell surface BsAb level and reduce cytokine release significantly. **A** The T cells were armed with different concentrates (0.32–200 nM) of anti-PSMA/anti-CD3 BsAbs. The surface BsAb level were detected by staining anti-His antibody and (**B)** measured as MFI by flow cytometry. **C** For testing the self-releasing cytokines of T cells, overactivated T cell (OKT3 co-cultured T cell), and BsAb-armed T cells, serial dilution of OKT3 or BsAbs were added to T cells for 1 h. After washed, the T cells, overactivated T cell or BsAb-armed T cells were incubated for 16 h at 37 °C. The supernatants were harvested for testing by commercial ELISA kits for human IL-2, TNF-α and IFN-γ. **D** The T cells arming with 0.4, 4, or 40 nM of each BsAb or OKT3 cultured T cell were applied at different ratios (effector cell/target cell ratios of 3:1, 5:1, or 10:1) and added to LNCaP. The anti-cancer efficiency was analyzed by cytotox 96 non-radioactive cytotoxicity assay kit. **E** The BsAb-armed T cells or BsAb mixed T cells were incubated with LNCaP for 16 h. The anti-cancer efficiency was analyzed by cytotox 96 non-radioactive cytotoxicity assay kit. Bar, SD; ***p < 0.001

**Fig. 5:**
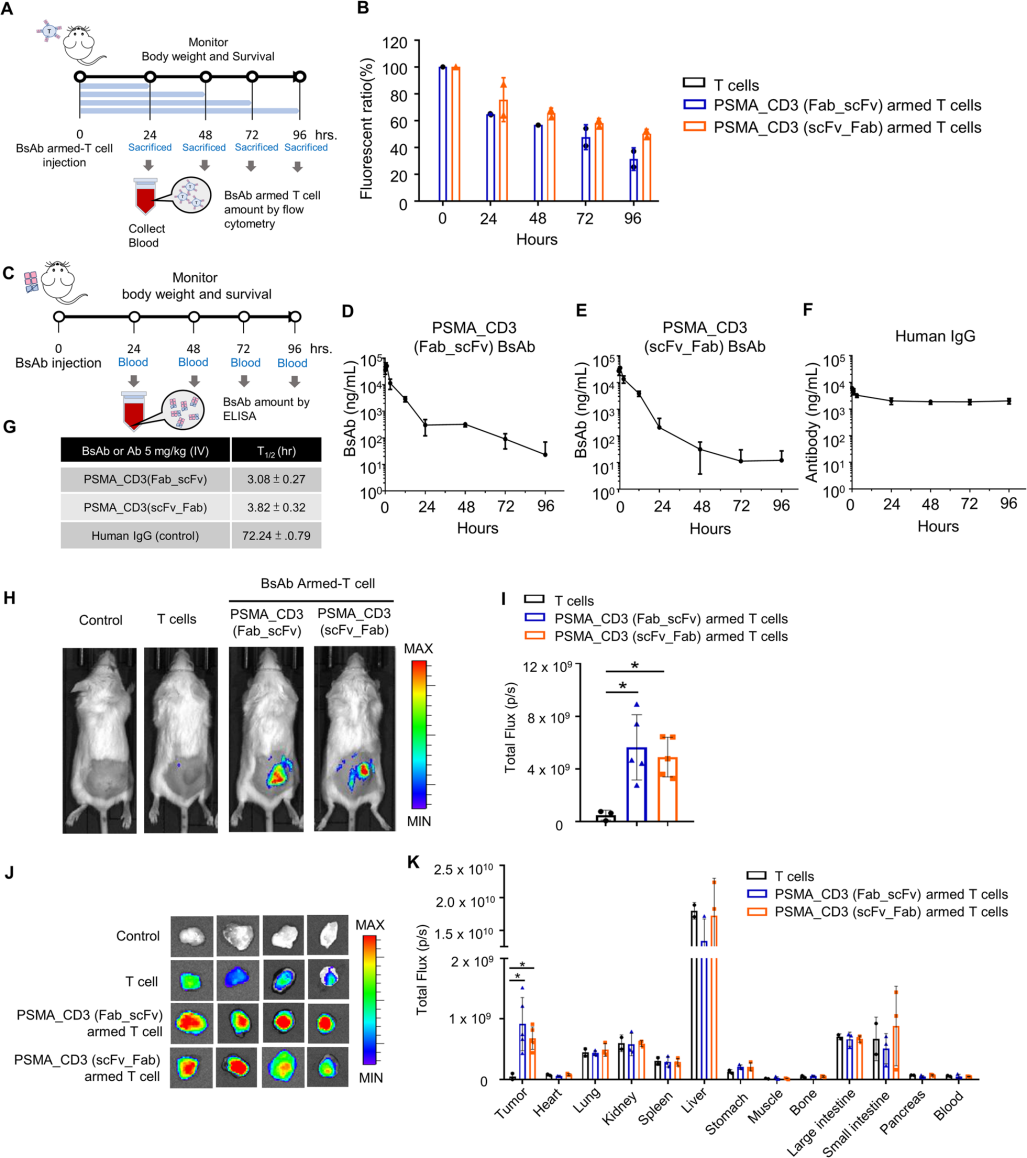
The pharmacokinetics and pharmacodynamics of BsAb-armed T cells. **A** Schematic of the pharmacokinetics experiment on BsAb-armed T cells. **B** 1 × 10^7^ T cells or BsAb-armed T cells were administered intravenously into SCID mice. Blood samples were collected at consecutive time points. The white blood cells were isolated from the blood by red blood cell lysis. The pharmacokinetics of BsAb-armed T cells were determined as CD3^+^ BsAb^+^ T cells and analyzed by flow cytometry. **C** Schematic of the BsAbs in vivo half-life analysis. The BsAb or human IgG was administered intravenously to C57BL/6 mice. Blood samples were collected at consecutive time points, and the serum concentrations of **D** anti-PSMA/anti-CD3 (Fab-scFv), **E** anti-PSMA/anti-CD3(scFv-Fab) BsAb, and **F** human IgG were quantified by sandwich ELISA. **G** The half-life of BsAbs in SCID mice. **H**–**K** The pharmacodynamics of BsAb-armed T cells: 1 × 10^7^ NIR-797-labeled T cells or BsAb-armed T cells were intravenously injected into LNCaP (100 mm^3^) bearing SCID mice. **H** The whole body was imaged 24 h post-injection with an IVIS spectrum imaging system. **I** The fluorescent intensity in tumors was analyzed with Living Image software (version 2.50). After mice were sacrificed 24 h post-injection, their **J** tumor and **K** organs were isolated for imaging with an IVIS spectrum imaging system to quantify their fluorescent signal. Bar, SD; *p < 0.05; **p < 0.01; ***p < 0.001

**Fig. 6:**
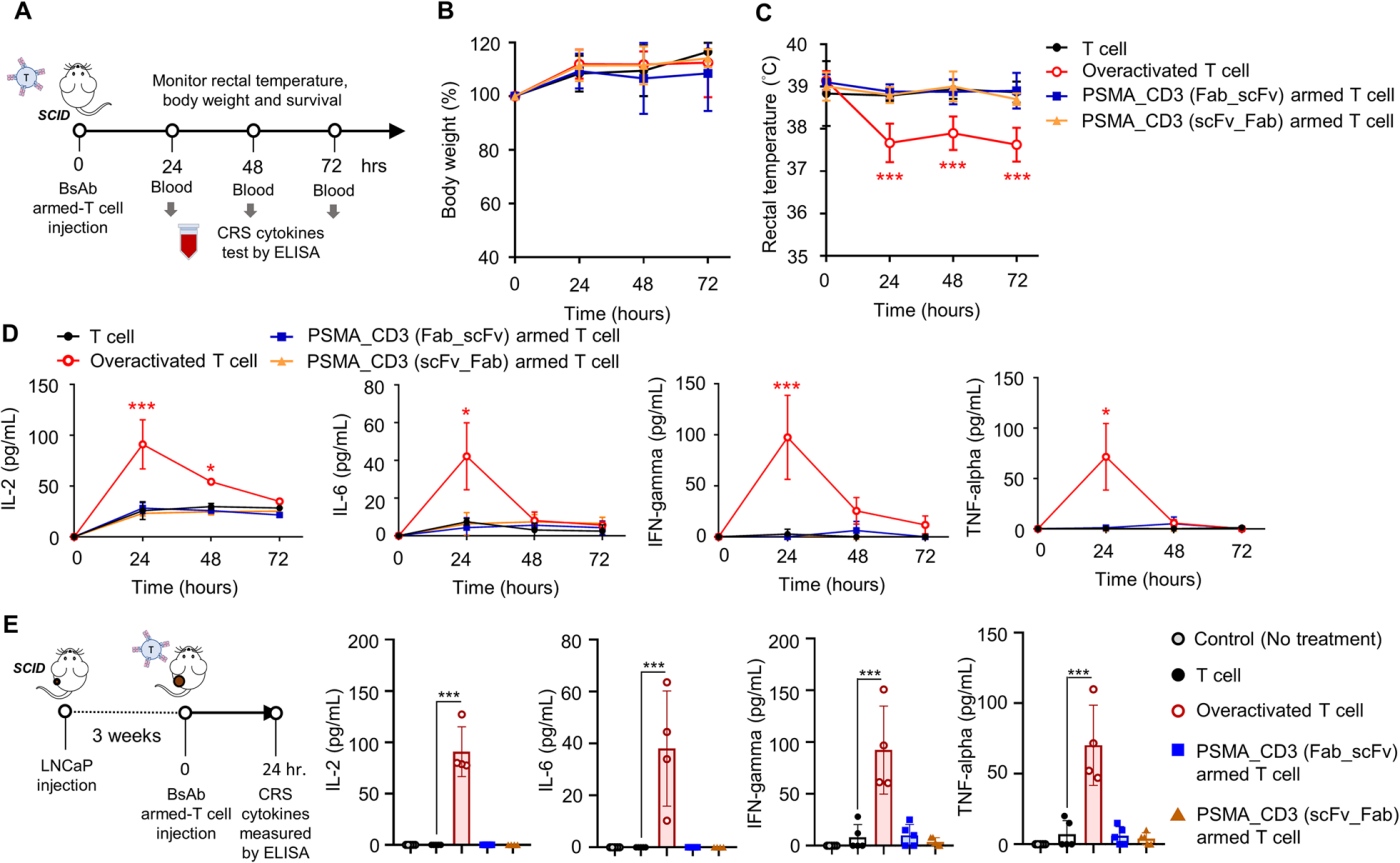
The in vivo cytokine release syndrome (CRS) evaluation of BsAb-armed T cells. **A** Schematic of in vivo CRS model of BsAb-armed T cells. The healthy SCID mice that received T cells, BsAb-armed T cells, or OKT3-induced overactivated T cells were measured **B** the body weight, **C** rectal temperatures, and **D** serum cytokine (IL-2, IL6, INF-γ, and TNF-α) levels at consecutive time points. **E** Serum cytokine (IL-2, IL6, INF-γ, and TNF-α) levels measured in tumor-bearing SCID mice treated with T cells, BsAb-armed T cells, or OKT3-induced overactivated T cells after 24 h. Bar, SD; *p < 0.05; **p < 0.01; ***p < 0.001

**Fig. 7:**
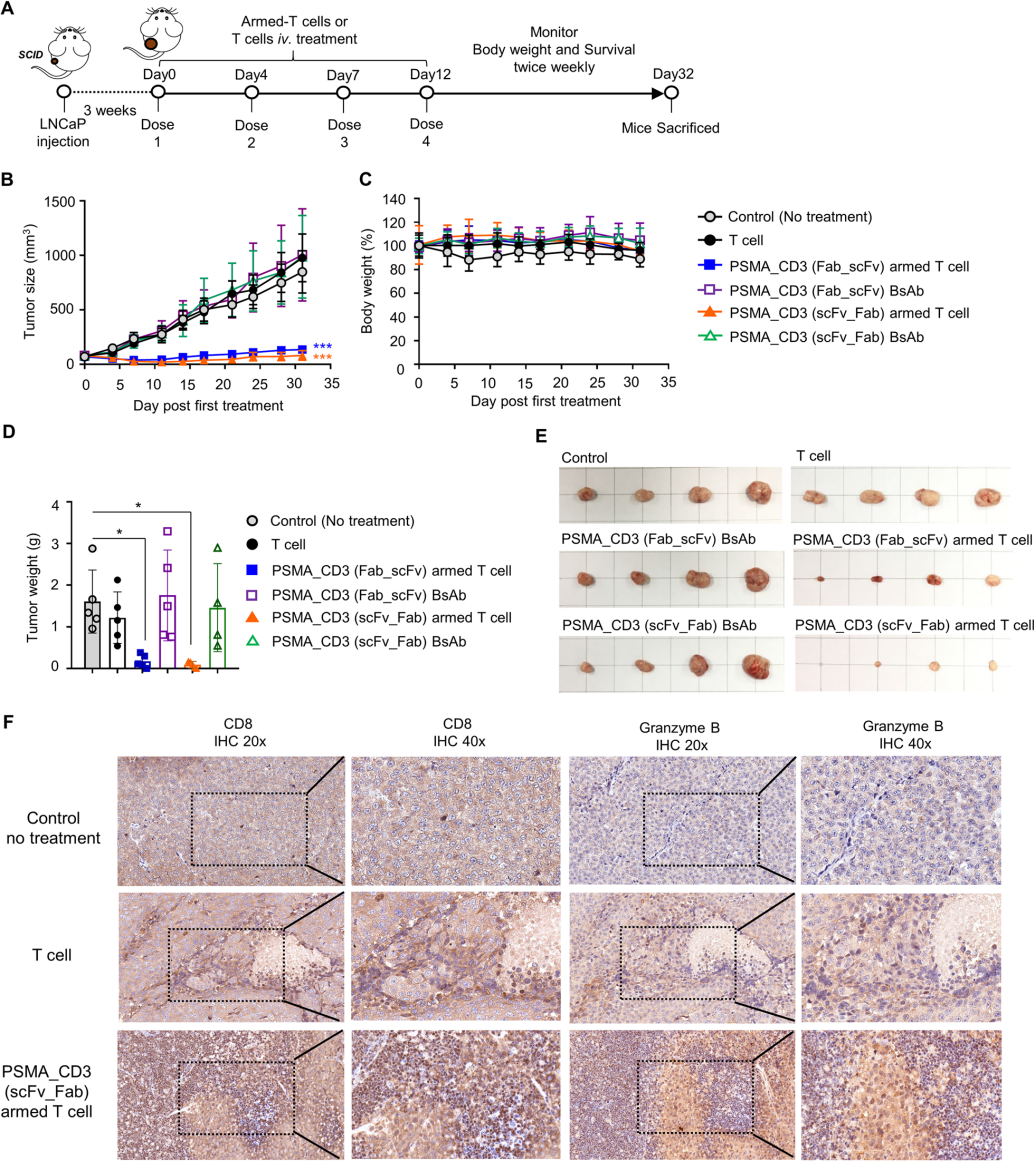
The in vivo therapeutic efficacy of BsAb-armed T cells. **A** A schematic showing the schedule of BsAb-armed T cell treatments. Here, 3 × 10^6^ T cells or BsAb-armed T cells were administered intravenously to (50 mm^3^) bearing SCID mice twice weekly for two weeks (n = 6). Their **B** tumor size and **C** body weight were observed. The mice were sacrificed on day 32, and **D**, **E** tumors were harvested from each group, weighed, and photographed. **F** SCID mice bearing established LNCaP (100 mm^3^) were intravenously injected with 5 × 10^6^ anti-PSMA/anti-CD3 (scFv-Fab) BsAb-armed T cells or OKT3-T cells twice weekly for 2 weeks. The tumors were isolated for IHC staining with anti-CD8 or anti-granzyme B antibodies. The images were taken at 20 × or 40 × magnification. Bar, SD; *p < 0.05; **p < 0.01; ***p < 0.001

The data were expressed as mean ± S.D. For Figs 5I, 6E and 7D, one-way ANOVA was used with Dunnett’s multiple comparisons. For Figs. 2, 3, 4, 5B, K, 6B–D, and 7B, C, two-way ANOVA was used with Dunnett’s or Bonferroni’s multiple comparisons. P values < 0.05 were considered statistically significant. Statistical analysis was performed by using Prism 8.0 software (GraphPad Software, La Jolla, CA, U.S.A.).

## Results

### Development and characterization of anti-cancer/anti-CD3 BsAbs

Although several anti-cancer/anti-CD3 BsAbs have been approved for clinical treatment, these BsAbs were initially designed to present with reduced CD3 binding affinity (> 10^−7^ M) to reduce the risks of unwanted T cell auto-activation after injection directly into the patient’s body [24–26]. Therefore, existing BsAbs cannot be used to stably “arm” the T cell surface due to their low CD3 binding affinities.

In this study, BsAbs with the ability to simultaneously bind to CD3 and tumor antigens (prostate-specific membrane antigen, PSMA) were generated by genetically fusing an anti-CD3 antibody with an anti-PSMA antibody. We designed four kinds of BsAb structures with different CD3 binding affinity: anti-cancer scFv/anti-CD3 scFv (the main design pattern of current clinical BsAbs), anti-cancer Fab/anti-CD3 scFv, anti-cancer scFv/anti-CD3 Fab, and anti-cancer antibody (with hole Fc)/anti-CD3 antibody (with knob Fc). All of these BsAbs were properly produced using the Expi-293 mammalian cell expression system and showed expected molecular sizes as visualized in both reducing and non-reducing SDS-PAGE (Additional file 11: figure S1). All different structures of anti-PSMA/anti-CD3 BsAb were specifically bonded to PSMA^+^ prostate cancer cells (LNCaP) and CD3^+^ T cells (Jurkat) but not PSMA^−^ CD3^−^ fibroblasts (NIH/3T3) **(**Fig. 1C**)**.

### One-step differentiation, expansion, and arming of T cells from PBMCs by BsAbs

Next, we tested whether the BsAbs could be used to differentiate, expand, and arm T cells from PBMCs. In clinical practice, a murine anti-human CD3 antibody (OKT3) is widely used to differentiate and expand T cells from PBMCs, so we used OKT3 as a comparison target here. Each of the BsAbs or OKT3 was added into a PBMC culture medium with IL-2. The differentiation and proliferation of CD3^+^ CD8^+^ T cells were assessed by flow cytometry. At day 7 (Additional file 11: figure S2) and day 14 **(**Fig. 2A**)**, the differentiation rate of CD3^+^ CD8^+^ T cells cultured by anti-PSMA/anti-CD3 (Fab-scFv) (day 7, 79.6%; day 14, 80.7%), anti-PSMA/anti-CD3 (scFv-Fab) (day 7, 91.7%; day 14.92%), or anti-PSMA/anti-CD3 (hole-knob) (day 7, 90.9%; day 14, 90.9%) was comparable to that when using the traditional OKT3 culture method (day 7, 83.8%; day 14.87%). However, culturing by anti-PSMA/anti-CD3 (scFv-scFv) exhibited a lower differentiation rate of CD3^+^ CD8^+^ T cells (day 7, 50.9%; day 14, 59.1%) that was only higher than that for PBMCs cultured by medium and IL-2 without OKT3 (day7, 35%; day 14, 40.9%). All antibody-cultured groups had superior cell expansion, total cell number, and cumulative population doubling level (CPDL) compared to the antibody-free group (Fig. 2C and Additional file 11: figure S3). The anti-PSMA/anti-CD3 BsAb groups (Fab-scFv, scFv-Fab, and hole-knob) had comparable CD3^+^ CD8^+^ T cell proliferation to the OKT3-cultured group (Fig. 2D). After T cells were cultured by the BsAbs, the four types of BsAbs were detected on the surface of T cells **(**Fig. 2B**)**. Besides, low levels of OKT3 were also detected on the surface of OKT3-cultured T cells **(**Fig. 2B**)**. These results indicate that the BsAb-based T cell culturing can efficiently differentiate and expand CD3^+^ CD8^+^ T cells from PBMCs and, moreover, endow these T cells with cancer specificity by arming BsAb on the T cell surfaces.

We further analyzed T cell activation marker CD25 and programmed cell death protein 1 (PD-1) markers on the surface of these T cells on day 7. T cells cultured with anti-PSMA/anti-CD3 (scFv-Fab) or anti-PSMA/anti-CD3 (hole-knob) had the highest CD25 expression levels, comparable to OKT3 cultured T cells. The anti-PSMA/anti-CD3 (scFv-scFv) cultured T cells had the lowest CD25 expression levels, similar to T cells cultured with medium alone (Additional file 11: figure **S**4A). In addition, the expression levels of exhaustion marker PD-1 were < 15% on the surface of all BsAb-cultured T cells. Among them, the anti-PSMA/anti-CD3 (scFv-Fab) cultured T cells had the lowest PD-1 expression level (3.4%) on T cell surface (Additional file 11: figure S4B). Besides, we also analyzed the surface expression levels of CD25 and PD-1 on BsAb-armed T cells before and after co-culturing with LNCaP or PC-3 cells. Before co-culturing with cancer cells, both OKT3 and anti-PSMA/anti-CD3 (scFv-Fab) BsAb-armed T cells expressed similar levels of surface CD25 (< 5%) and PD-1 (< 2%). After co-culturing with LNCaP but not PC-3 cells, the anti-PSMA/anti-CD3 BsAb-armed T cells had significantly higher surface CD25 levels (86.3%) and moderate surface PD-1 levels (42.2%). Additionally, surface expression of CD25 and PD-1 on OKT3 T cells did not change significantly after co-culturing with LNCaP or PC-3 cells (Additional file 11: figure S5).

### In vitro tumor killing efficacy of BsAb-armed T cells

Next, we compared the tumor killing ability of T cells cultured by anti-PSMA/anti-CD3 BsAbs (BsAb-armed T cells) or by OKT3 (OKT3-T cells). All of the anti-PSMA BsAb-armed T cells showed a dramatically higher killing ability against LNCaP cells than the OKT3-T cells did (Fig. 3A). After being co-cultured with LNCaP cells, all of the anti-PSMA BsAb-armed T cells released a higher amount of cytokines (IL-2, INF-γ, and TNF-α) and cytotoxins (perforin and granzyme B) than the OKT3-T cells (Fig. 3B). Additionally, the tumor killing ability and cytokine/cytotoxin releasing of each of the anti-PSMA BsAb-armed T cell groups against PC-3 were similar to those of the OKT3-T cell group (Fig. 3A and C).

To further examine the tumor targeting and killing efficacy of anti-PSMA BsAb-armed T cells, the cellular dynamics and behavior of anti-PSMA BsAb-armed T cells co-cultured withLNCaP cells were observed using real-time optical microscopy. All of the anti-PSMA BsAb-armed T cells rapidly accumulated at LNCaP cell areas and expressed effective tumor killing ability. In contrast, the OKT3-T cells, moving aimlessly around the cancer cells, lacked the ability to target and kill the cancer cells (Fig. 3D, E; Additional file 1: Movie 1, Additional file 2: Movie 2, Additional file 3: Movie 3, Additional file 4: Movie 4, Additional file 5: Movie 5). In addition, all anti-PSMA BsAb-armed T cells and OKT3-T cells did not attack PC-3 cells (Additional file 6: Movie 6, Additional file 7: Movie 7, Additional file 8: Movie 8, Additional file 9: Movie 9, Additional file 10: Movie 10, Additional file 11: figure S6), indicating that anti-PSMA BsAb-armed T cells have high tumor-killing specificity and excellent safety. Moreover, the BsAb loading on the surface of the BsAb-armed T cells could be further increased by incubating the armed T cells with additional amounts of BsAb (Fig. 4A, B). The levels of self-releasing cytokines (IL-2, TNF-α, and INF-γ) of T cells incubated with up to 43.7 nM of BsAb were significantly lower than those of OKT3-cultured T cells (Fig. 4C), indicating that the BsAb does not stimulate excessive T cell autoactivation. According to western blot results, the maximum dose of anti-PSMA/anti-CD3 (scFv-Fab) on the surface of T cells was considered to be approximately 34.8 ± 4.8 μg/10^9^ T cells (Additional file 11: Figure S7 and table S1). We further demonstrated that the tumor killing efficiency of T cells armed with 6–100% of the maximum anti-PSMA/anti-CD3 (Fab-scFv) loading amount or with 17% to 100% of the maximum anti-PSMA/anti-CD3 (scFv-Fab) loading amount on their surfaces was significantly higher than that of OKT3-T cells in killing LNCaP cells (Fig. 4D, Additional file 11: table S2-S3). Importantly, the anti-PSMA BsAb-armed T cells (armed with anti-PSMA/anti-CD3 [Fab-scFv] or anti-PSMA/anti-CD3 [scFv-Fab] structures) showed a higher killing efficacy against LNCaP cells (Fig. 4E) and a lower T cell auto-activation risk (Additional file 11: figure S8-S9) compared with T cells mixed with different quantities of free BsAbs.

### The kinetics of BsAb-armed T cells in SCID mice

Since BsAbs are armed on the surface of T cells by non-covalent bonding, the in vivo retention time of BsAbs on the surface of T cells will determine the anti-cancer effect of BsAb-armed T cells. To test the retention time, we injected separately two kinds of anti-PSMA/anti-CD3 (Fab-scFv and scFv-Fab) BsAb-armed T cells into SCID mice, and then analyzed the residual amount of BsAbs on the surface of the T cells in the blood at different time points (Fig. 5A). The half-life of anti-PSMA/anti-CD3 BsAb (Fab-scFv) and anti-PSMA/anti-CD3 BsAb (scFv-Fab) retained on T cells were 83.8 and 135.4 h, respectively (Fig. 5B). At 96 h, the residual amounts of the BsAbs on T cells were 39.1 ± 10.5% and 62.9 ± 4.1%, respectively (Additional file 11: table S4). These results indicate that anti-PSMA/anti-CD3 BsAb (scFv-Fab) with intact anti-CD3 Fab structure has a better CD3 binding affinity and can persist in binding on T cell surfaces for a longer period of time. In addition, we also examined the serum half-life of BsAbs (Fab-scFv and scFv-Fab) and human IgG in a mouse model (Fig. 5C). The half-life of human IgG, Fab-scFv, and scFv-Fab in mice were 72.24 ± 0.79, 3.08 ± 0.27, and 3.82 ± 0.32 h, respectively (Fig. 5D–G). Therefore, the BsAbs’ (Fab-scFv or scFv-Fab) lack of an Fc domain makes them disappear more rapidly once they leave the T cell surface, effectively reducing the possibility of unwanted interactions with normal cells.

### The biodistrbution of BsAb-armed T cells in tumor-bearing mice

To evaluate the in vivo tumor targeting and biodistribution of BsAb-armed T cells, we separately labeled a near-infrared fluorescent probe (NIR-797) on various anti-PSMA/anti-CD3 BsAb-armed T cells or conventional T cells (OKT3-T cells), and then compared the tumor targeting efficiency and biodistribution of these T cells in mice bearing LNCaP tumors by using an optical imaging system. Figure 5H shows that the anti-PSMA/anti-CD3 BsAb-armed T cells accumulated at the LNCaP tumor areas by the 24th hour after the intravenous administration. The cumulative amounts of anti-PSMA/anti-CD3 (scFv-Fab and Fab-scFv) BsAb-armed T cells in the LNCaP tumor areas were, respectively, 10.1 and 11.7 times higher than that of OKT3-T cells (Fig. 5I). Next, we harvested all of the organs and tumors and quantified the fluorescent intensity using an optical imaging system. The cumulative amounts of anti-PSMA/anti-CD3 (scFv-Fab and Fab-scFv) BsAb-armed T cells in the tumor tissues were, respectively, 13.7 and 18.4 times higher than that of the OKT3- T cells (Fig. 5J–K). Importantly, the localization of BsAb-armed T cells in each normal organ was similar to that of the OKT3-T cell group.

### The in vivo safty of BsAb-armed T cells in tumor-bearing or health mice model

We further analyzed the rectal temperature change, cytokine release, and serum toxicity of heathy SCID mice after being treated with anti-PSMA/anti-CD3 (Fab-scFv or scFv-Fab) BsAb-armed T cells or overactive T cells (induced by additional intravenous injectiob of OKT3) (Fig. 6A). There was no significant change in body weight (Fig. 6B) or organ weight (Additional file 11: figure S10) in each group at 72 h post-injection. However, the rectal temperature of the overactive T cell group was 1.5 °C lower than those of the other groups (Fig. 6C). Moreover, the levels of inflammatory cytokines, including IL-2, IL6, INF-γ, and TNF-α, in the overactive T cell group were significantly higher than the levels in the other groups at 24 h (Fig. 6D). The liver enzyme indices, including ALT and AST, in the overactive T cell group were also significantly higher than those indices in the other groups at 48 h (Additional file 11: figure S11). Importantly, the levels of inflammatory cytokines did not increase in LNCaP tumor-bearing SCID mice after treatment with anti-PSMA BsAb-armed T cells (Fig. 6E). Collectively, anti-PSMA BsAb-armed T cells did not induce any obvious serum toxicity in the examined animal models.

### In vivo anti-tumor efficacy of BsAb-armed T cells

Finally, we evaluated the in vivo anti-tumor efficacy of two types of anti-PSMA/anti-CD3 (Fab-scFv and scFv-Fab) BsAb-armed T cells, using SCID mice bearing LNCaP tumors (Fig. 7A). On day 32 post-treatment, the T cell group (tumor size: 976 ± 437.3 mm^3^) and the anti-PSMA/anti-CD3 BsAb groups (Fab-scFv and scFv-Fab) (tumor sizes: 1004 ± 876.33 mm^3^ and 987 ± 654.4 mm^3^, respectively) were not effective in inhibiting tumor growth (control group, 878 ± 386.6 mm^3^). In contrast, both anti-PSMA/anti-CD3 (Fab-scFv and scFv-Fab) BsAb-armed T cell groups had excellent tumor suppressive effects and even eliminated tumors (Fig. 7B). Among these, the scFv-Fab BsAb-armed T cell group was the most effective (tumor size: Fab-scFv BsAb-armed T cell, 135.4 ± 70.16 mm^3^; scFv-Fab BsAb-armed T cell, 82.2 ± 88.7 mm^3^) in tumor elimination. In addition, there was no significant change in body weight in each group over the course of treatment (Fig. 7C), indicating that the BsAb-armed T cell treatment did not cause additional toxicity to the bodies of the mice. We further harvested and weighed tumors from each group. The anti-PSMA/anti-CD3 (scFv-Fab) BsAb-armed T cell group had the smallest tumor weight (0.09 ± 0.07 g), followed by the anti-PSMA/anti-CD3 (Fab-scFv) BsAb-armed T cell group (0.17 ± 0.14 g). The tumor weights of the other groups were not significantly different from that of the control (medium) group (1.61 ± 0.68 g) **(**Fig. 7D, E**)**. Furthermore, we compared the infiltration and anti-tumor activity of anti-PSMA/anti-CD3 (scFv-Fab) BsAb-armed T cells and OKT3-T cells in the tumor area by CD8 and granzyme B immunohistochemical staining, respectively. Figure 7F shows that the anti-PSMA BsAb-armed T cell treated group had much higher CD8^+^ T cell infiltration in the tumor area than the OKT3-T cell treated group. Interestingly, in the OKT3-T cell treated group, while a small amount of CD8^+^ T cells infiltrated the tumor area, these T cells did not actively release granzyme B. In contrast, the anti-PSMA BsAb-armed-T cells released a large amount of granzyme B in the tumor area. This result indicates that the anti-PSMA BsAb-armed T cells have much better tumor-infiltrating and tumor-killing abilities in the solid tumor area than normal T cells.

## Discussion

In this study, we developed a novel BsAb-based culture platform for cancer-specific T cells. By using anti-cancer/anti-CD3 BsAbs with higher CD3 binding affinity, especially those with the scFv-Fab structure, to culture human PBMCs, cancer-specific BsAb-armed T cells can be rapidly generated with a purity of over 90% in 7 days. The in vitro and in vivo experiments demonstrated highly efficient targeting and cytotoxicity of BsAb-armed T cells against human cancer cells (PSMA^+^ prostate cancer) without severe cellular cytokine release or other significant toxicities. The BsAb-armed T cell platform is a fast-culturing and low-cost method for generating highly pure and non-genetically engineered cancer-specific T cells.

The success of CAR T cells in the treatment of hematological malignancies has made uses of such cells the fastest-growing class of cancer-specific T cell therapies in recent years. However, CAR-T cell therapies still face many challenges in solid tumors due to the immunosuppressive tumor microenvironment. Malignant tumors often highly express programmed death-ligand 1 (PD-L1) as an immunosuppressive molecule targeting PD-1 on T cells, causing T cell exhaustion and reducing the T cells’ tumor-killing ability [27]. Therefore, new approaches for genetically modifying CAR-T cells to prevent the PD-1/PD-L1 interaction are under development. For example, Dr. Marasco and colleagues engineered a CAR-T cell that constitutively secretes anti-PD-L1 antibodies to block the PD-L1 molecules on the tumor surface [28]. Dr. Marson and colleagues reported the CRISPR/Cas9-mediated PD-1 disruption of CAR-T cells. These strategies focus on improving the anti-tumor effects of CAR-T cells by preventing PD-1/PD-L1-mediated immunosuppression [29]. In this study, the T cells cultured with BsAbs, especially anti-PSMA scFv/anti-CD3 Fab, had low PD-1 expression on their surface (Additional file 11: figure S4) and a strong therapeutic effect against PD-L1 expressing human prostate tumors (LNCaP; Additional file 11: figure S12) in SCID mouse models.

Moreover, the generation of current CAR T cells is still based on retroviral and lentiviral gene transfer, the use of which poses a risk of T cell carcinogenesis [30]. Even though there are safer non-viral vectors and nuclear gene editing technologies available, many obstacles to the clinical application of these technologies are still present. Taking CRISPR/Cas9 techniques as an example, recent studies have found that cells with normal functional p53 are resistant to the genetic editing of CRISPR/Cas9, while successfully edited cells tend to undergo cell death or exhibit p53 mutation, which also raises oncogenic risk [31, 32]. A more critical factor in the clinical outcomes of CAR-T cell therapies is the transduction efficiency of the CAR gene. Although the gene transfer efficacy of the lentiviral system is superior to that of CRISPR/Cas9 techniques, the average purity of CAR- T cells in two clinical products (Kymriah and Yescarta) is lower than 40% [33]. In addition, laborious and time-consuming protocols (the need for lentiviral transfection, the screening of CAR-T cells, the amplification of CAR-T cells, and the characterization of viral particles in CAR-T cells) make the manufacturing of CAR-T cells take almost 30 days before the product is ready to be administered back into the patient. In contrast, the anti-cancer/anti-CD3 BsAb-based cultivation of PBMCs generates cancer-specific T cells with a purity of more than 90% in 7 days and without genetic modification. Not only could these T cells continuously proliferate ex vivo for more than 20 days, but they also grew at a rate comparable to that of the traditional OKT3-based T cells. On average, after being cultured with the anti-PSMA/anti-CD3 (scFv-Fab) and IL-2 for 20 days, 10^8^ PBMCs could be converted into more than 10^11^ effector BsAb-armed T cells. Moreover, these BsAb-armed T cells, once contacted with tumors, can trigger TCR/CD3 downstream signaling to convert T cells into a highly activated state with high surface CD25 expression and moderate surface PD-1 expression (Additional file 11: figure S5), thereby promoting T cell proliferation and the releasing of cytotoxins (perforin and granzyme B) from T cells to kill tumors (Fig. 3B). As a result, we believe that the BsAb-based method can easily be scaled up for industrial manufacturing and can provide cancer-specific T cells abundantly enough for single or repeated administration for each patient.

Anti-cancer/anti-CD3 bispecific antibodies have been studied for decades and used in solid and hematologic cancer therapies. BsAbs injected into cancer patients can bind to cancer cells and CD3^+^ T cells and directly evoke the cancer-killing activity of T cells via CD3 signaling without MHC mediation [34, 35]. This strategy can overcome cancer cells’ immune escape by reducing or abolishing their MHC expression. The first anti-cancer/anti-CD3 bispecific antibody approved by the EMA was Catumaxomab [36, 37]. This BsAb with a complete Fc domain had an increased in vivo half-life [38] and induced antibody-dependent cell-mediated (ADCC) and complement-dependent (CDC) cytotoxicities against tumors. However, ADCC and CDC can also harm T cells bound by Fc-containing BsAbs.

Next, bispecific T cell engagers (BiTEs) lacking the Fc domain were developed and showed therapeutic effects in clinical cancer treatment, including blinatumomab (AMG110), AMG330, and AMG596. However, the lack of an Fc domain gives BiTEs an extremely short in vivo half-life. Patients receiving blinatumomab required continuous infusion for 28 days to maintain its therapeutic concentration [39], which is inconvenient and prone to inducing cytokine release syndrome (CRS) or neurotoxicity [39–41]. Additionally, without knowing the distribution and number of T cells in each patient’s body, predicting how many BiTE doses are required to optimally direct T cells to the tumor area is difficult. The anti-CD3 domain of these injected BiTEs might also bind immunosuppressive T regulatory (Treg) or proinflammatory T helper 17 (Th17) cells in patients’ bodies, which might interferes with the anti-cancer efficacy of effector T cells and even induce severe CRS [42, 43]. Unlike BiTEs, the BsAb-armed T cell platform can directly arm the anti-cancer/anti-CD3 BsAbs on the surface of effector T cells but not Th17 or Treg cells during the T cell culturing process. In addition, the anti-PSMA/anti-CD3 (scFv-Fab) BsAb amount on the surfaces of BsAb-armed T cells was only 34.8 ± 4.8 μg for 10^9^ T cells in one treatment, much lower than the BiTE dose directly injected into patients [44, 45]. Overall, the BsAb-armed T cell platform can reduce BsAb amounts used for each treatment and accurately modify the BsAb to effector T cells, which combines the therapeutic advantages of anti-cancer/anti-CD3 bispecific antibodies and adoptive T therapies in cancer treatment and effectively reduces body toxicities effectively.

Several strategies using ex vivo armed T cells with BsAb have been rapidly developed for cancer treatment. For example, Dr. Rambaldi and colleagues applied blinatumomab (anti-CD19 scFv/anti-CD3 scFv structure) to ex vivo cultured effector T cells (named BET cells) from patients with B cell leukemia. This strategy effectively expanded the effector T cells and eliminated B cell leukemia in the isolated PBMCs during the culturing process [46]. However, blinatumomab’s relatively low CD3 binding affinity (10^−7^ M) makes it challenging to stably arm the surface of BET cells, resulting in insufficient cytotoxic effects against cancer cells. Therefore, additional blinatumomab intravenous infusion of blinatumomab becomes necessary in BET cell-based therapeutic strategies [46]. Dr. Lum and colleagues applied a chemical method using Traut’s reagent and sulfo-SMCC to crosslink monoclonal anti-cancer and anti-CD3 antibodies to arm ex vivo expanded cytotoxic T cells (named BAT cells) [47–49]. Several BAT cell products have proven safe in multiple clinical trials without severe CRS, neurotoxicity, or organ toxicities, including anti-GD2/anti-CD3 (hu3F8 linked with mouse OKT3) [50], anti-HER2/anti-CD3 (trastuzumab linked with mouse OKT3) [51], and anti-EGFR/anti-CD3 (cetuximab linked with mouse OKT3) [52]. However, chemically synthesized BsAbs often show an inconsistent antibody orientation that might reduce the anti-cancer ability of armed T cells and affect reproducibility in product quality and manufacturing [53, 54]. Dr. Cheung and colleagues developed a recombinant bispecific antibody with an IgG-[L]-scFv structure (anti-CD3 scFv fused to the C-terminus of the anti-tumor IgG light chain) attached to ex vivo armed T cells (named EATs), which had effective anti-tumor abilities for neuroblastoma and osteosarcoma in vitro and in vivo [55, 56]. The anti-CD3 structure of IgG-[L]-scFv uses the bivalent CD3 scFv, providing a higher CD3 binding affinity and enhancing CD3-associated T cell activation to increase the tumor-killing ability. However, recent studies reported that bivalent CD3 binding might induce antigen-independent auto-activation of T cells, causing unwanted cytokine release [57–59]. Therefore, our anti-PSMA/anti-CD3 BsAb designs avoided bivalent CD3 binding and focused on developing the monovalent CD3-binding domain (scFv or Fab) to avoid inducing auto-activation of T cells without binding to tumor cells.

We found that the structure of anti-cancer/anti-CD3 BsAbs determined the efficiency of T cell differentiation from PBMCs and the effective period of the BsAb-armed T cells for killing tumors in vivo. The proportions of CD8^+^ T cells differentiated by BsAbs with the anti-CD3 scFv structure (anti-PSMA/anti-CD3 [scFv-scFv and Fab-scFv]) were significantly lower than those cultured by BsAbs with the anti-CD3 Fab structure (anti-PSMA/anti-CD3 [scFv-Fab and hole-knob]) and traditional OKT3 (Fig. 2A and Additional file 11: figure S2). Besides, T cells cultured with the anti-CD3 Fab-based BsAbs expressed higher levels of activation marker CD25 and lower levels of exhaustion marker PD-1 on their surface than the anti-CD3 scFv-based BsAbs (Additional file 11: figure S4). The binding of anti-CD3 antibodies to T cells can directly modulate TCR/CD3 downstream signaling, further triggering T cell proliferation and anti-tumor activity. We speculate that the low affinity of anti-CD3 scFv, which is at least tenfold weaker than anti-CD3 Fab and whole antibodies (Additional file 11: figure S13), means that it does not trigger CD3 signal transduction sufficiently to stimulate T cell expansion and activation. Rambaldi et al. reported similar results, where blinatumomab expanded CD8^+^ T cells with a differentiation rate of 44.4 ± 28.3% [46]. In addition, many studies have shown that OKT3 scFv does not induce T cell proliferation and cytokine secretion as effectively as intact OKT3 antibody [60, 61]. The in vivo experiments in this study also demonstrated that the BsAbs containing an anti-CD3 Fab have longer retention times on the surfaces of T cells (Fig. 5B), resulting in better anti-cancer efficacy in human tumor-bearing SCID mice models (Fig. 7).

In addition to directly using BsAb to culture and develop cancer-specific T cells (BsAb-armed T cells), we can accurately control the total amount of BsAb armed on the surfaces of T cells by adding extra BsAb to T cells after culturing. Thus, it is easy to generate and customize armed T cells with different amounts (10% to 100%) of BsAb on their surface (Fig. 4A). Other T cell culturing methods, such as those utilizing CD3/CD28 binding beads [62–64], can also be applied in this arming strategy to quickly expand BsAb-armed T cells. More importantly, the T cells armed with 10% to 100% of the maximum anti-cancer/anti-CD3 (scFv-Fab) loading amount exhibited similar tumor killing ability. The low BsAb density threshold allows for longer therapeutic efficacy of armed T cells, which is a critical advantage for in vivo cancer treatment. After T cells armed with 100% BsAb were injected into tumor-bearing mice, 50% of the anti-cancer/anti-CD3 (scFv-Fab) remained on the T cell surfaces to provide tumor targeting and killing ability for these T cells at the 135^th^ hour post-injection. As described above, BsAb-armed T cells can thus maintain high efficacy in terms of their ability to kill tumors in vivo over a long period of time.

## Conclusions

Compared to genetic engineering T cell technologies, which require complicated and long-term preparation processes, the BsAb-armed T cell technology provides an extremely simplified method for producing cancer-specific T cells with a purity of over 90%, with the process only requiring the co-culturing of PBMCs with anti-cancer/anti-CD3 (scFv-Fab) BsAb and IL-2 for 7 days. Additionally, easily controlling the surface amounts of BsAb on T cells allows us to generate customized BsAb-armed T cells with higher therapeutic efficacy and lower CRS risk for individual patients. This technology will provide an immediate T cell immunotherapy to cancer patients who are dying of rapidly progressing disease.

## Supplementary Information

**Additional file 1: Movie 1.** Time-lapse video created using live-cell images of T cells co-cultured with LNCaP.

**Additional file 2: Movie 2.** Time-lapse video created using live-cell images of anti-PSMA/anti-CD3 (Fab-scFv) BsAb-armed T cells co-cultured with LNCaP.

**Additional file 3: Movie 3.** Time-lapse video created using live-cell images of anti-PSMA/anti-CD (scFv-Fab) 3BsAb-armed T cells co-cultured with LNCaP.

**Additional file 4: Movie 4.** Time-lapse video created using live-cell images of anti-PSMA/anti-CD3 (scFv-scFv) BsAb-armed T cells co-cultured with LNCaP.

**Additional file 5: Movie 5.** Time-lapse video created using live-cell images of anti-PSMA/anti-CD3 (hole-knob) BsAb-armed T cells co-cultured with LNCaP.

**Additional file 6: Movie 6.** Time-lapse video created using live-cell images of T cells co-cultured with PC-3.

**Additional file 7: Movie 7.** Time-lapse video created using live-cell images of anti-PSMA/anti-CD3 (Fab-scFv) BsAb-armed T cells co-cultured with PC-3.

**Additional file 8: Movie 8.** Time-lapse video created using live-cell images of anti-PSMA/anti-CD3 (scFv-Fab) BsAb-armed T cells co-cultured with PC-3.

**Additional file 9: Movie 9.** Time-lapse video created using live-cell images of anti-PSMA/anti-CD3 (scFv-scFv) BsAb-armed T cells co-cultured with PC-3.

**Additional file 10: Movie 10.** Time-lapse video created using live-cell images of anti-PSMA/anti-CD3 (hole-knob) BsAb-armed T cells co-cultured with PC-3.

**Additional file 11: Table S1.** Maximum dose of anti-PSMA/anti-CD3 (scFv-Fab) BsAb on T cell surface. **Table S2.** The cancer killing efficiency levels of anti-PSMA/anti-CD3 (Fab-scFv) BsAb-armed T cells armed with different levels of BsAb. **Table S3.** The cancer killing efficiency of anti-PSMA/anti-CD3 (scFv-Fab) BsAb-armed T cells arming with different level BsAb. **Table S4.** The residual amount of anti-PSMA/anti-CD3 BsAbs on the surface of T cells. **Figure S1.** Production and analysis of recombinant anti-PSMA/anti-CD3 BsAbs. **Figure S2.** The ex vivo differentiation of T cells using anti-PSMA/anti-CD3 BsAbs on day 7. **Figure S3.** The proliferation rate of ex vivo expanded T cells or BsAb-armed T cells. **Figure S4.** The CD25 and PD-1 expression levels of T cells cultured with anti-PSMA/anti-CD3 BsAbs on day 7. **Figure S5.** The CD25 and PD-1 expression levels of anti-PSMA/anti-CD3 BsAb-armed T cells before and after co-culturing with prostate cancer cells. **Figure S6.** Time-lapse live video microscopy of anti-PSMA BsAb-armed-T cells co-cultured with PC-3. **Figure S7.** The amount of anti-PSMA/anti-CD3 (scFv-Fab) BsAb-armed on T cell surface. **Figure S8.** T cell auto-activation of anti-PSMA/anti-CD3 (Fab-scFv) BsAb-armed T cells and T cell mixed with anti-PSMA/anti-CD3 (Fab-scFv) BsAbs. **Figure S9.** T cell auto-activation of anti-PSMA/anti-CD3 (scFv-Fab) BsAb-armed T cells and T cell mixed with anti-PSMA/anti-CD3 (scFv-Fab) BsAbs. **Figure S10.** The organ weight of anti-PSMA/anti-CD3 BsAb-armed T cells treated SCID mice. **Figure S11.** The liver index of T cells or BsAb-armed T cells treated SCID mice. **Figure S12.** PD-L1 expression levels in LNCaP cell-line. **Figure S13.** The human CD3 binding affinity of anti-PSMA/anti-CD3 (scFv-Fab) BsAbs.

## Data Availability

Raw data generated and analyzed for the article are included in the manuscript and its additional information files, or are available on reasonable request.

## Abbreviations

CD3: Cluster of differentiation 3
PBMC: Peripheral blood mononuclear cell
BsAb: Bispecific antibody
TNFα: Tumor necrosis factor α
IFNγ: Interferon γ
SCID: Severe combined immunodeficiency
CPDL: Cumulative population doubling level
CD4: Cluster of differentiation 4
CD8: Cluster of differentiation 8
CD28: Cluster of differentiation 28
CD19: Cluster of differentiation 19
IL-2: Interleukin 2
IL-6: Interleukin 6
TCR: T cell receptor
MHC: Major histocompatibility complex
CAR: Chimeric antigen receptor
FDA: Food and Drug Administration
EMA: European Medicines Agency
Fab: Fragment antigen-binding
ScFv: Single-chain fragment variable
Fc: Fragment crystallizable
PSMA: Prostate-specific membrane antigen
His epitope: Histidine epitope
HA epitope: Hemagglutinin epitope
SDS-PAGE: Sodium dodecyl sulfate polyacrylamide gel electrophoresis
PD-1: Programmed death-1
ELISA: Enzyme-linked immunosorbent assay
ALT: Alanine transaminase
AST: Aspartate transaminase
ADCC: Antibody-dependent cell-mediated cytotoxicity
CDC: Complement-dependent cytotoxicity
CRS: Cytokine release syndrome
Th17 cell: T helper 17 cell
