# Supplementary material for: A non-genetic engineering platform for rapidly generating and expanding cancer-specific armed T cells
Source: J Biomed Sci. 2023 May 31;30:35. doi: 10.1186/s12929-023-00929-z (PMC10230823; doi:10.1186/s12929-023-00929-z)
Supplement: Supplementary file 11 — Additional file 11: Table S1. Maximum dose of anti-PSMA/anti-CD3 (scFv-Fab) BsAb on T cell surface. Table S2. The cancer killing efficiency levels of anti-PSMA/anti-CD3 (Fab-scFv) BsAb-armed T cells armed with different levels of BsAb. Table S3. The cancer killing efficiency of anti-PSMA/anti-CD3 (scFv-Fab) BsAb-armed T cells arming with different level BsAb. Table S4. The residual amount of anti-PSMA/anti-CD3 BsAbs on the surface of T cells. Figure S1. Production and analysis of recombinant anti-PSMA/anti-CD3 BsAbs. Figure S2. The ex vivo differentiation of T cells using anti-PSMA/anti-CD3 BsAbs on day 7. Figure S3. The proliferation rate of ex vivo expanded T cells or BsAb-armed T cells. Figure S4. The CD25 and PD-1 expression levels of T cells cultured with anti-PSMA/anti-CD3 BsAbs on day 7. Figure S5. The CD25 and PD-1 expression levels of anti-PSMA/anti-CD3 BsAb-armed T cells before and after co-culturing with prostate cancer cells. Figure S6. Time-lapse live video microscopy of anti-PSMA BsAb-armed-T cells co-cultured with PC-3. Figure S7. The amount of anti-PSMA/anti-CD3 (scFv-Fab) BsAb-armed on T cell surface. Figure S8. T cell auto-activation of anti-PSMA/anti-CD3 (Fab-scFv) BsAb-armed T cells and T cell mixed with anti-PSMA/anti-CD3 (Fab-scFv) BsAbs. Figure S9. T cell auto-activation of anti-PSMA/anti-CD3 (scFv-Fab) BsAb-armed T cells and T cell mixed with anti-PSMA/anti-CD3 (scFv-Fab) BsAbs. Figure S10. The organ weight of anti-PSMA/anti-CD3 BsAb-armed T cells treated SCID mice. Figure S11. The liver index of T cells or BsAb-armed T cells treated SCID mice. Figure S12. PD-L1 expression levels in LNCaP cell-line. Figure S13. The human CD3 binding affinity of anti-PSMA/anti-CD3 (scFv-Fab) BsAbs. [file 12929_2023_929_MOESM11_ESM.docx]

***Additional file***

**A Non-Genetic Engineering Platform for Rapidly Generating and Expanding Cancer-Specific Armed T Cells**

Yi-Jou Chen^1^, Michael Chen^1^, Tian-Lu Cheng^2, 3, 4^, Yi-Shan Tsai^5^, Chang-Hung Wang^6^, Che-Yi Chen^6^, Tung-Yun Wu^6^, Shey-Cherng Tzou^7^, Kai-Hung Wang^8^, Jing-Jy Cheng^9^, An-Pei Kao^10^, Shyr-Yi Lin^11, 12, 13,^ *, Kuo-Hsiang Chuang^1, 2, 5, 6, 14, 15, 16,^ *

^1^Graduate Institute of Pharmacognosy, Taipei Medical University, Taipei, Taiwan

^2^Drug Development and Value Creation Research Center, Kaohsiung Medical University, Kaohsiung, Taiwan

^3^Graduate Institute of Medicine, College of Medicine, Kaohsiung Medical University, Kaohsiung, Taiwan

^4^Department of Biomedical Science and Environmental Biology, Kaohsiung Medical University, Kaohsiung, Taiwan

^5^Master Program in Clinical Genomics and Proteomics, Taipei Medical University, Taipei, Taiwan

^6^ Ph.D. Program in Clinical Drug Development of Herbal Medicine, Taipei Medical University, Taipei, Taiwan

^7^Departmet of Biological Science and Technology, National Yang Ming Chiao Tung University, Hsinchu, Taiwan

^8^Center for Reproductive Medicine, Kuo General Hospital, Tainan, Taiwan

^9^National Research Institute of Chinese Medicine, Ministry of Health and Welfare, Taipei, Taiwan

^10^CytoArm Co., Ltd, Taipei, Taiwan

^11^Division of Gastroenterology and Hepatology, Department of Internal Medicine, Taipei Medical University Hospital, Taipei, Taiwan.

^12^Department of General Medicine, School of Medicine, College of Medicine, Taipei Medical University, Taipei, Taiwan

^13^TMU Research Center of Cancer Translational Medicine, Taipei Medical University, Taipei, Taiwan

^14^Traditional Herbal Medicine Research Center of Taipei Medical University Hospital, Taipei, Taiwan

^15^Ph.D Program in Biotechnology Research and Development, Taipei Medical University, Taipei, Taiwan

^16^The Ph.D. Program of Translational Medicine, Taipei Medical University, Taipei, Taiwan

The authors declare no potential conflicts of interest.

* Corresponding Author:

Dr. Kuo-Hsiang Chuang

Graduate Institute of Pharmacognosy, Taipei Medical University,

250 Wu-Hsing Street, Taipei, TAIWAN

Phone: +886-2-27361663-6163

E-mail: [khchuang@tmu.edu.tw](mailto:khchuang@tmu.edu.tw)

Dr. Shyr-Yi Lin

Division of Gastroenterology and Hepatology, Department of Internal Medicine, Taipei Medical University Hospital.

252 Wu-Hsing Street, Taipei, TAIWAN

E-mail: [sylin@tmu.edu.tw](mailto:sylin@tmu.edu.tw)

**Additional file includes:**

**Supplemental Figures**

Supplemental Figure 1. Production and analysis of recombinant anti-PSMA/anti-CD3 BsAbs.

Supplemental Figure 2. The *ex vivo* differentiation of T cells using anti-PSMA/anti-CD3 BsAbs on day 7.

Supplemental Figure 3. The proliferation rate of *ex vivo* expanded T cells or BsAb-armed T cells.

Supplemental Figure 4. The CD25 and PD-1 expression levels of T cells cultured with anti-PSMA/anti-CD3 BsAbs on day 7.

Supplemental Figure 5. The CD25 and PD-1 expression levels of anti-PSMA/anti-CD3 BsAb-armed T cells before and after co-culturing with prostate cancer cells.

Supplemental Figure 6. Time-lapse live video microscopy of anti-PSMA BsAb-armed-T cells co-cultured with PC-3.

Supplemental Figure 7. The amount of anti-PSMA/anti-CD3 (scFv-Fab) BsAb-armed on T cell surface.

Supplemental Figure 8. T cell auto-activation of anti-PSMA/anti-CD3 (Fab-scFv) BsAb-armed T cells and T cell mixed with anti-PSMA/anti-CD3 (Fab-scFv) BsAbs.

Supplemental Figure 9. T cell auto-activation of anti-PSMA/anti-CD3 (scFv-Fab) BsAb-armed T cells and T cell mixed with anti-PSMA/anti-CD3 (scFv-Fab) BsAbs.

Supplemental Figure 10. The organ weight of anti-PSMA/anti-CD3 BsAb-armed T cells treated SCID mice.

Supplemental Figure 11. The liver index of T cells or BsAb-armed T cells treated SCID mice.

Supplemental Figure 12. PD-L1 expression levels in LNCaP cell-line.

Supplemental Figure 13. The human CD3 binding affinity of anti-PSMA/anti-CD3 BsAbs.

**Supplemental Tables**

Supplementa1 Table 1. Maximum dose of anti-PSMA/anti-CD3 (scFv-Fab) BsAb on T cell surface.

Supplemental Table 2. The cancer killing efficiency levels of anti-PSMA/anti-CD3 (Fab-scFv) BsAb-armed T cells armed with different levels of BsAb.

Supplemental Table 3. The cancer killing efficiency of anti-PSMA/anti-CD3 (scFv-Fab) BsAb-armed T cells arming with different level BsAb.

Supplemental Table 4. The residual amount of anti-PSMA/anti-CD3 BsAbs on the surface of T cells.

**Supplemental Movies**

Supplemental Movie 1. Time-lapse video created using live-cell images of T cells co-cultured with LNCaP.

Supplemental Movie 2. Time-lapse video created using live-cell images of anti-PSMA/anti-CD3 (Fab-scFv) BsAb-armed T cells co-cultured with LNCaP.

Supplemental Movie 3. Time-lapse video created using live-cell images of anti-PSMA/anti-CD3 (scFv-Fab) BsAb-armed T cells co-cultured with LNCaP.

Supplemental Movie 4. Time-lapse video created using live-cell images of anti-PSMA/anti-CD3 (scFv-scFv) BsAb-armed T cells co-cultured with LNCaP.

Supplemental Movie 5. Time-lapse video created using live-cell images of anti-PSMA/anti-CD3 (hole-knob) BsAb-armed T cells co-cultured with LNCaP.

Supplemental Movie 6. Time-lapse video created using live-cell images of T cells co-cultured with PC-3.

Supplemental Movie 7. Time-lapse video created using live-cell images of anti-PSMA/anti-CD3 (Fab-scFv) BsAb-armed T cells co-cultured with PC-3.

Supplemental Movie 8. Time-lapse video created using live-cell images of anti-PSMA/anti-CD3 (scFv-Fab) BsAb-armed T cells co-cultured with PC-3.

Supplemental Movie 9. Time-lapse video created using live-cell images of anti-PSMA/anti-CD3 (scFv-scFv) BsAb-armed T cells co-cultured with PC-3.

Supplemental Movie 10. Time-lapse video created using live-cell images of anti-PSMA/anti-CD3 (hole-knob) BsAb-armed T cells co-cultured with PC-3.

**SupplementaL Figures**


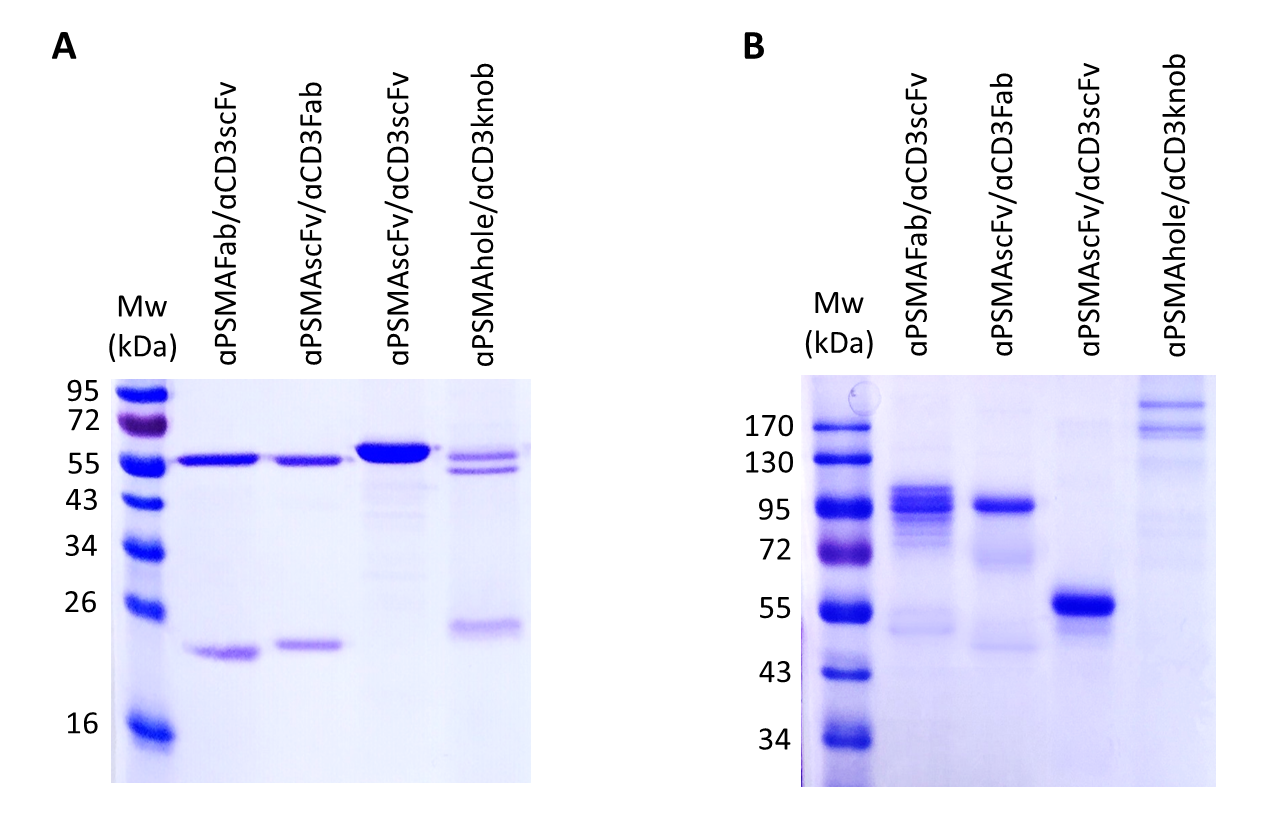


**Supplemental Figure 1.** **Production and analysis of recombinant anti-PSMA/anti-CD3 BsAbs.** (**A**) Reducing and (**B**) non-reducing SDS-PAGE show the coomassie blue staining of anti-PSMA/anti-CD3 BsAbs (left to right, Fab-scFv, scFv-Fab, scFv-scFv and knob-hole).

**
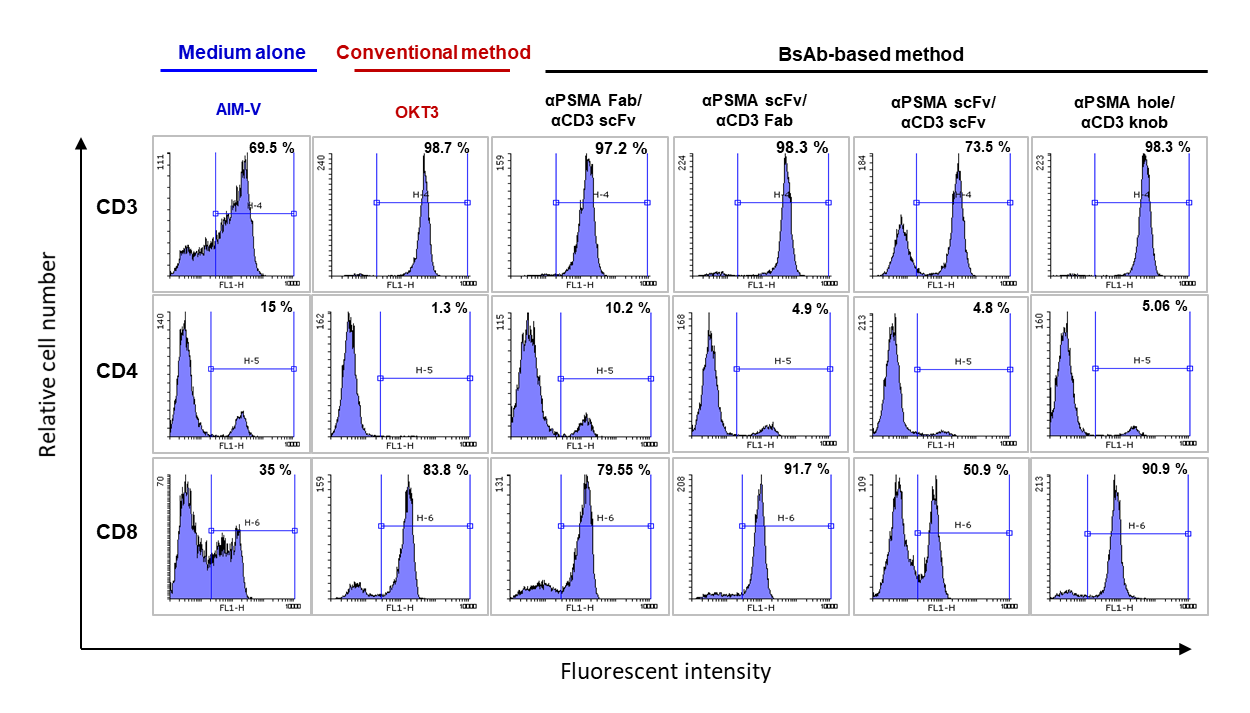
**

**Supplemental Figure 2. The *ex vivo* differentiation of T cells using anti-PSMA/anti-CD3 BsAbs on day 7.** Human PBMCs were isolated from the blood of healthy donors and co-cultured with different BsAbs or traditional mouse anti-CD3 antibodies (OKT3). On day 7, the T cell population of each group was analyzed by FITC conjugated anti-CD3 antibody, FITC conjugated anti-CD4 antibody, and FITC conjugated anti-CD8 antibody combined with flow cytometry.


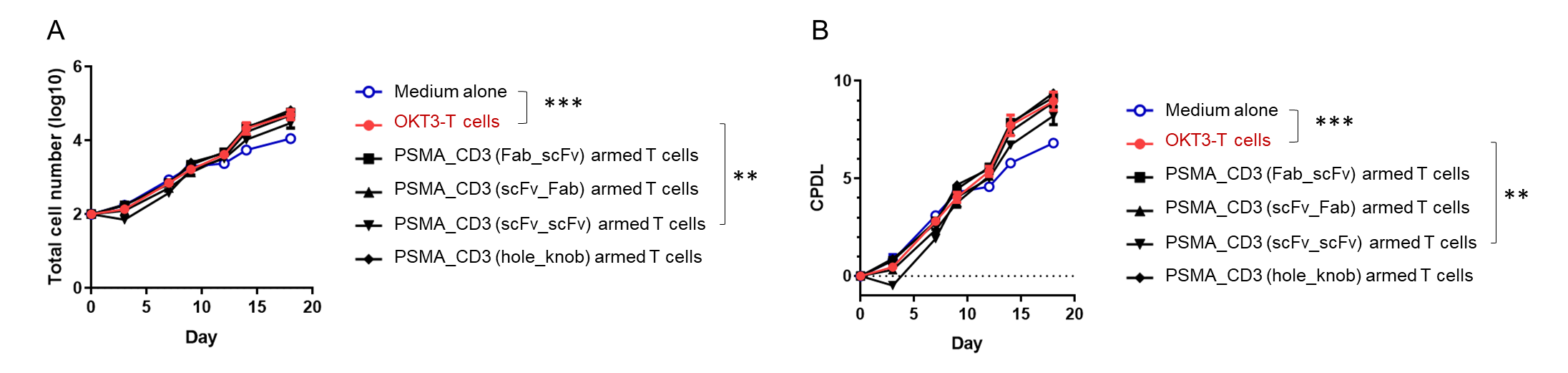


**Supplemental Figure 3**. **The proliferation rate of *ex vivo* expanded T cells or BsAb-armed T cells.** (A) Cell growth rates over 20 days of T cell culturing. Total live cell numbers were counted using the trypan blue staining method, which excludes dead cells. (B) Cell doubling rates over 20 days of T cell culturing. The doubling rates were evaluated by cumulative population-doubling level (CPDL). The population doubling level was calculated using the following equation: PDL=3.32 × log(*N*_harvest_/*N*_seeding_), where *N*_seeding_ is the initial seeded live cell number, and *N*_harvest_ is the total live cell number at harvest.


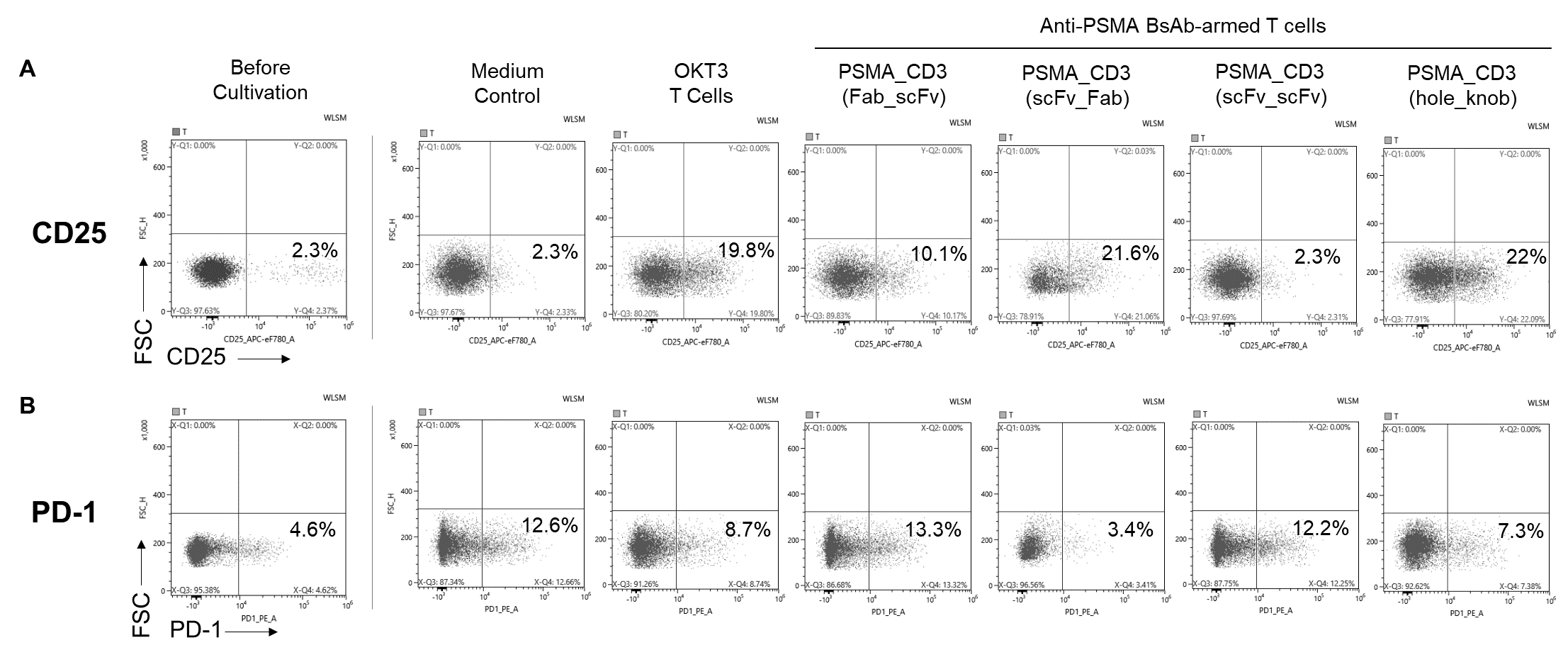


**Supplemental Figure 4. The CD25 and PD-1 expression levels of T cells cultured with anti-PSMA/anti-CD3 BsAbs on day 7.** Human PBMCs were isolated from the blood of healthy subjects and co-cultured with various BsAbs or OKT3. On day 7, the surface expression of (A) CD25 and (B) PD-1 on each T cell group was analyzed by APC-eFluor 780-conjugated anti-CD25 antibody, and PE-conjugated anti-PD-1 antibody combined with flow cytometry.


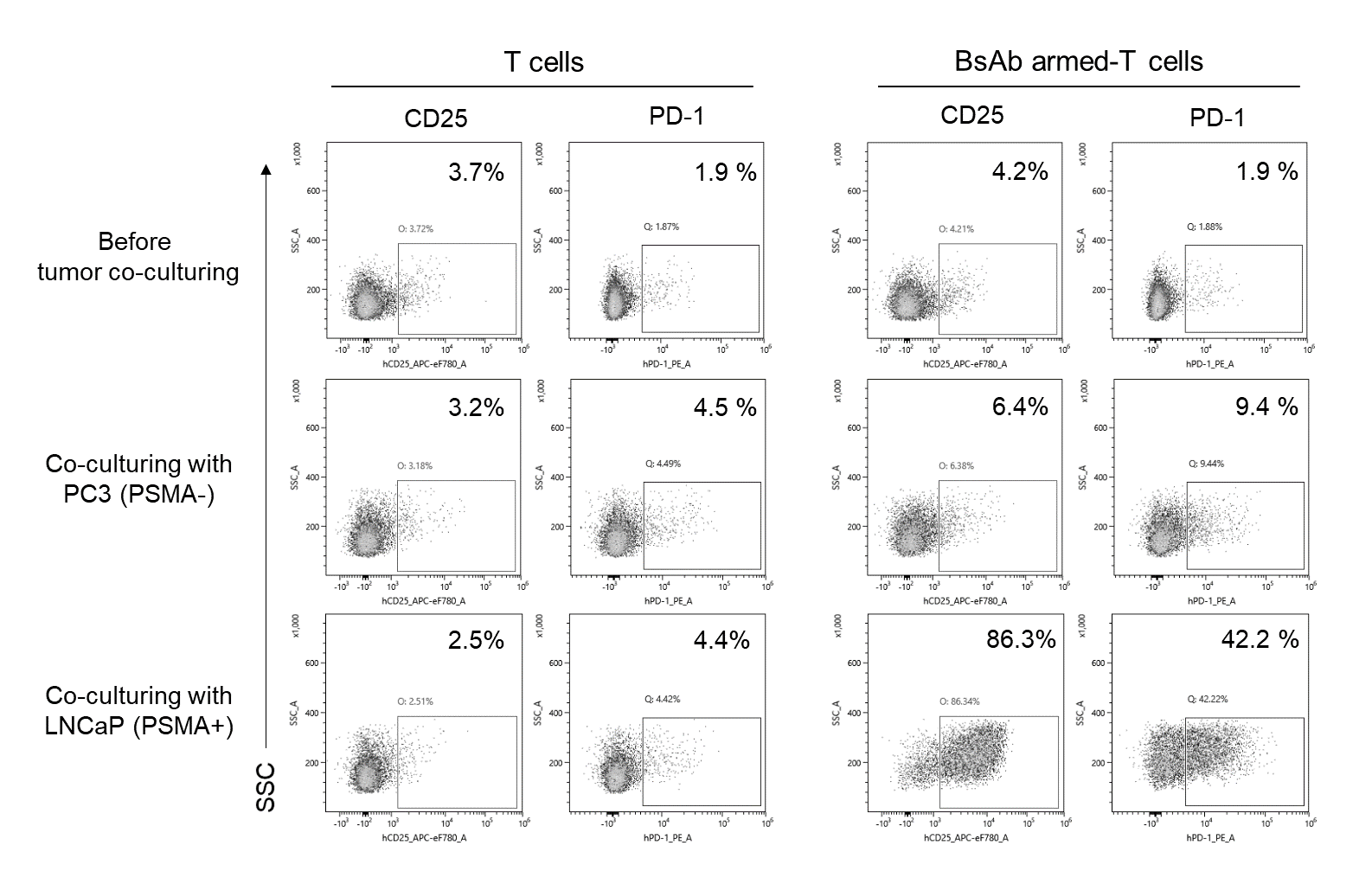


**Supplemental Figure 5.** **The CD25 and PD-1 expression levels of anti-PSMA/anti-CD3 BsAb-armed T cells before and after co-culturing with prostate cancer cells.** The anti-PSMA/anti-CD3 BsAb (scFv-Fab)-armed T cells and OKT3-T cells were co-cultured with PSMA^+^ (LNCaP) or PSMA^−^ (PC-3) cancer cells for 16 hours. Each T cell group’s surface expression of CD25 and PD-1 was assessed by flow cytometry using APC-eFluor 780-conjugated anti-CD25 and PE-conjugated anti-PD-1 antibodies.


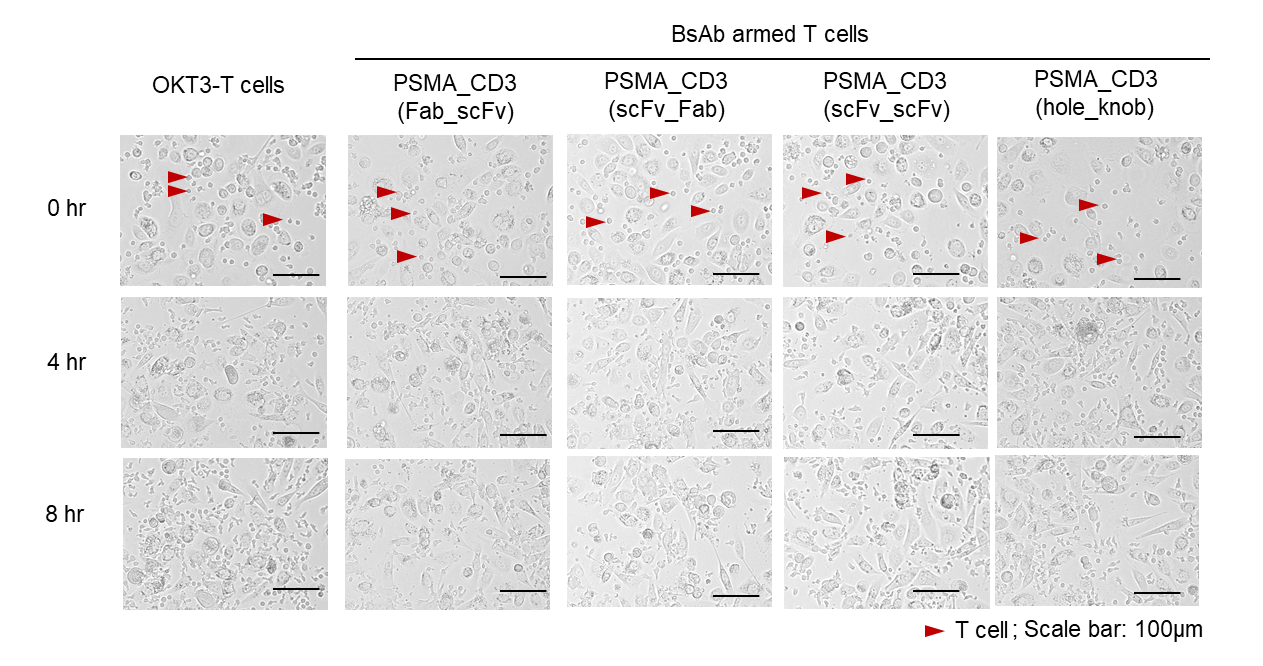


**Supplemental Figure 6. Time-lapse live video microscopy of anti-PSMA BsAb-armed T cells co-cultured with PC-3.** PC-3 cells were seeded at a density of 2×10^4^ cells/well in 96-well plates and incubated at 37°C. After 24 hours, the T cells or anti-PSMA BsAb-armed T cells were added at a 2:1 E:T ratio and incubated at 37°C. The images were taken at 5-minute intervals for 8 hours using a Cytation 3 multi-mode reader (Bio-Tek, Winooski, VT, USA). The image analysis was performed using the Movie Maker software (Windows, USA).

**
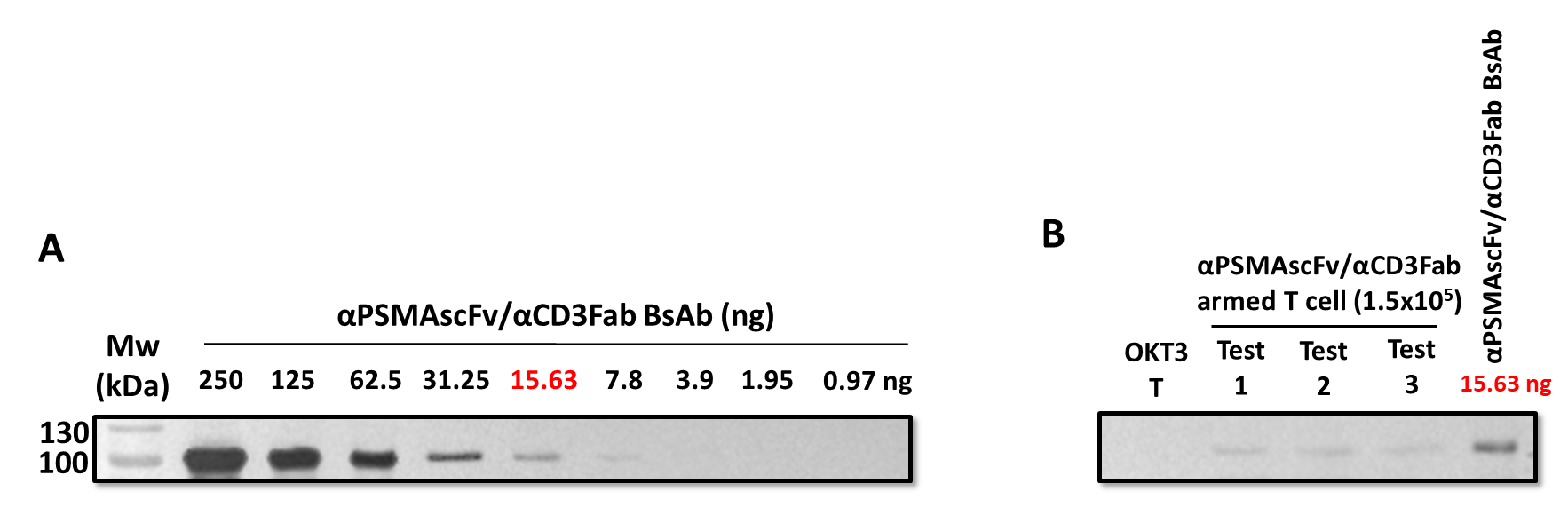
**

**Supplemental Figure 7.** **The amount of anti-PSMA/anti-CD3 (scFv-Fab) BsAb-armed on T cell surface.** (**A**) the non-reducing Western blot data show the signal of serial diluted anti-PSMA scFv/anti-CD3 Fab BsAb detected by HRP conjugated goat anti-human IgG Fab. (**B**) the western blot data show the amount of BsAb binding on T cell surface.


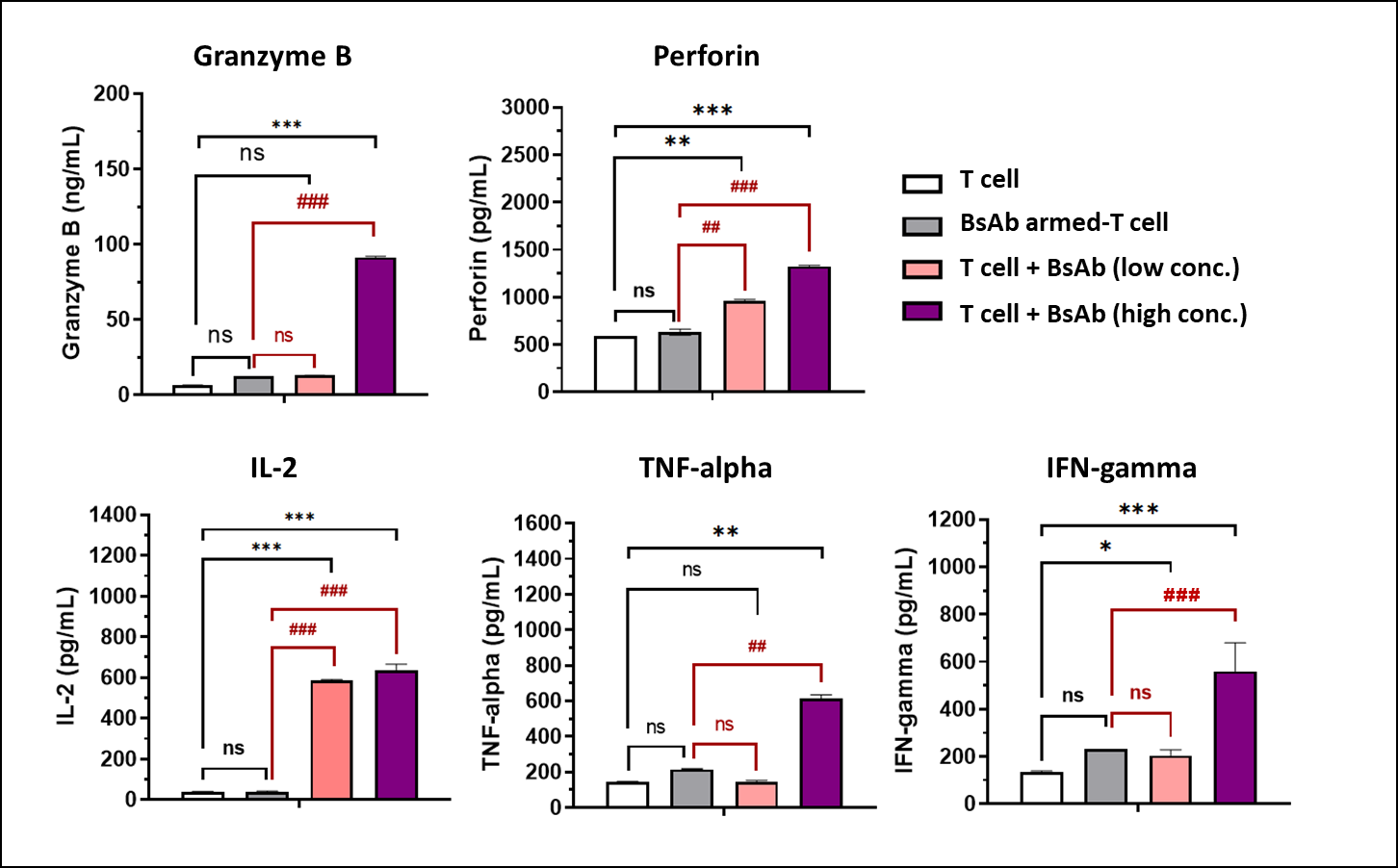


**Supplemental Figure 8.** **T cell auto-activation of anti-PSMA/anti-CD3 (Fab-scFv) BsAb-armed T cells and T cell mixed with anti-PSMA/anti-CD3 (Fab-scFv) BsAbs.** anti-PSMA/anti-CD3 (Fab-scFv) armed T cells and T cells mixed with different concentrates of anti-PSMA/anti-CD3 (Fab-scFv) BsAbs were incubated for 18 hours, and then the supernatants of each group were harvested to analyze the secretion of cytotoxins or cytokines by ELISAs. Bar, SD. ns, not-significance. *, P<0.05. **, P<0.01. ***, P<0.001. #, P<0.05. ##, P<0.01. ###, P<0.001.

**
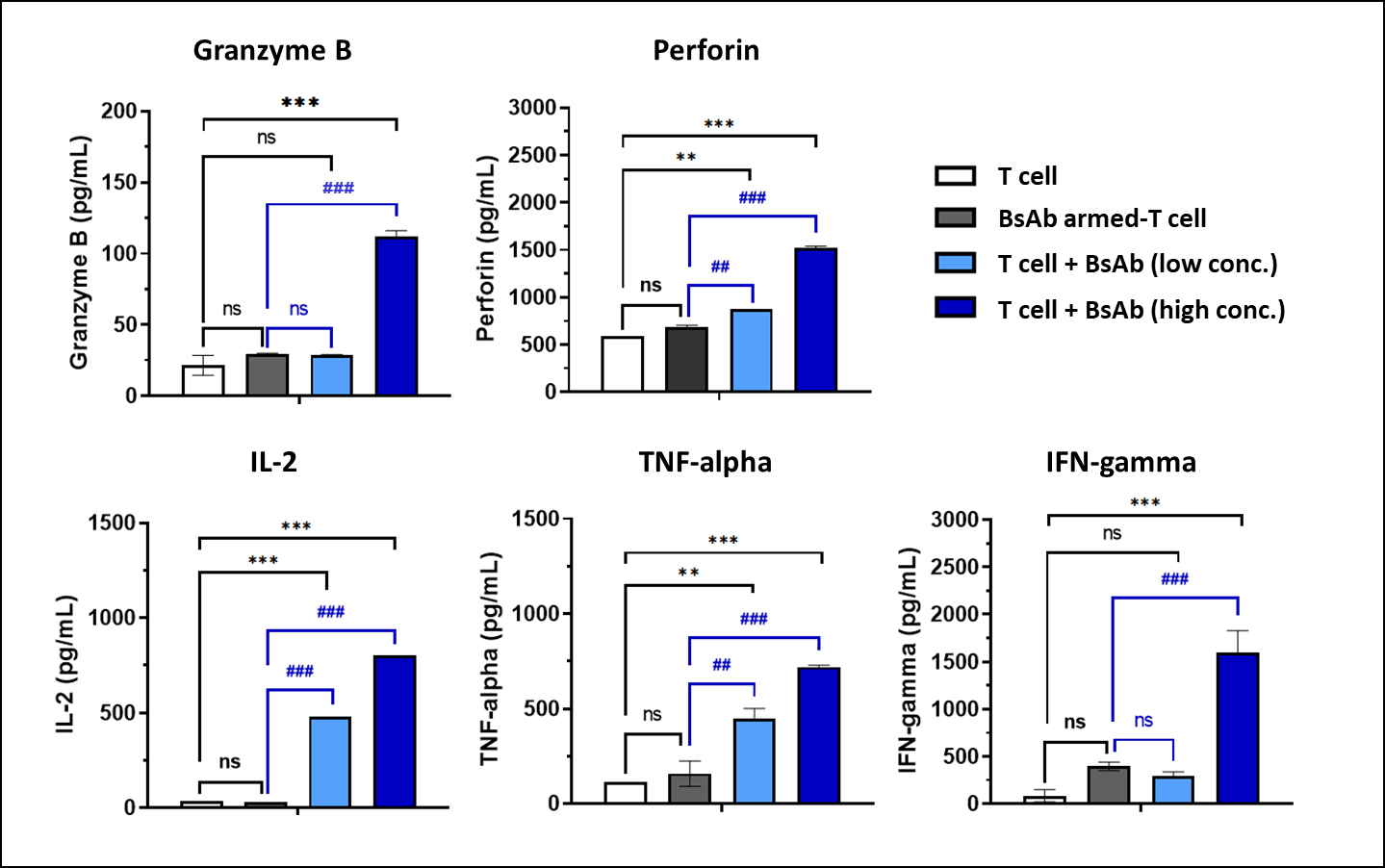
**

**Supplemental Figure 9.** **T cell auto-activation of anti-PSMA/anti-CD3 (scFv-Fab) BsAb-armed T cells and T cell mixed with anti-PSMA/anti-CD3 (scFv-Fab) BsAbs.** anti-PSMA/anti-CD3 (scFv-Fab) armed T cells and T cells mixed with different concentrates of anti-PSMA/anti-CD3 (scFv-Fab) BsAbs were incubated for 18 hours, and then the supernatants of each group were harvested to analyze the secretion of cytotoxins or cytokines by ELISAs. Bar, SD. ns, not-significance. *, P<0.05. **, P<0.01. ***, P<0.001. #, P<0.05. ##, P<0.01. ###, P<0.001.

**
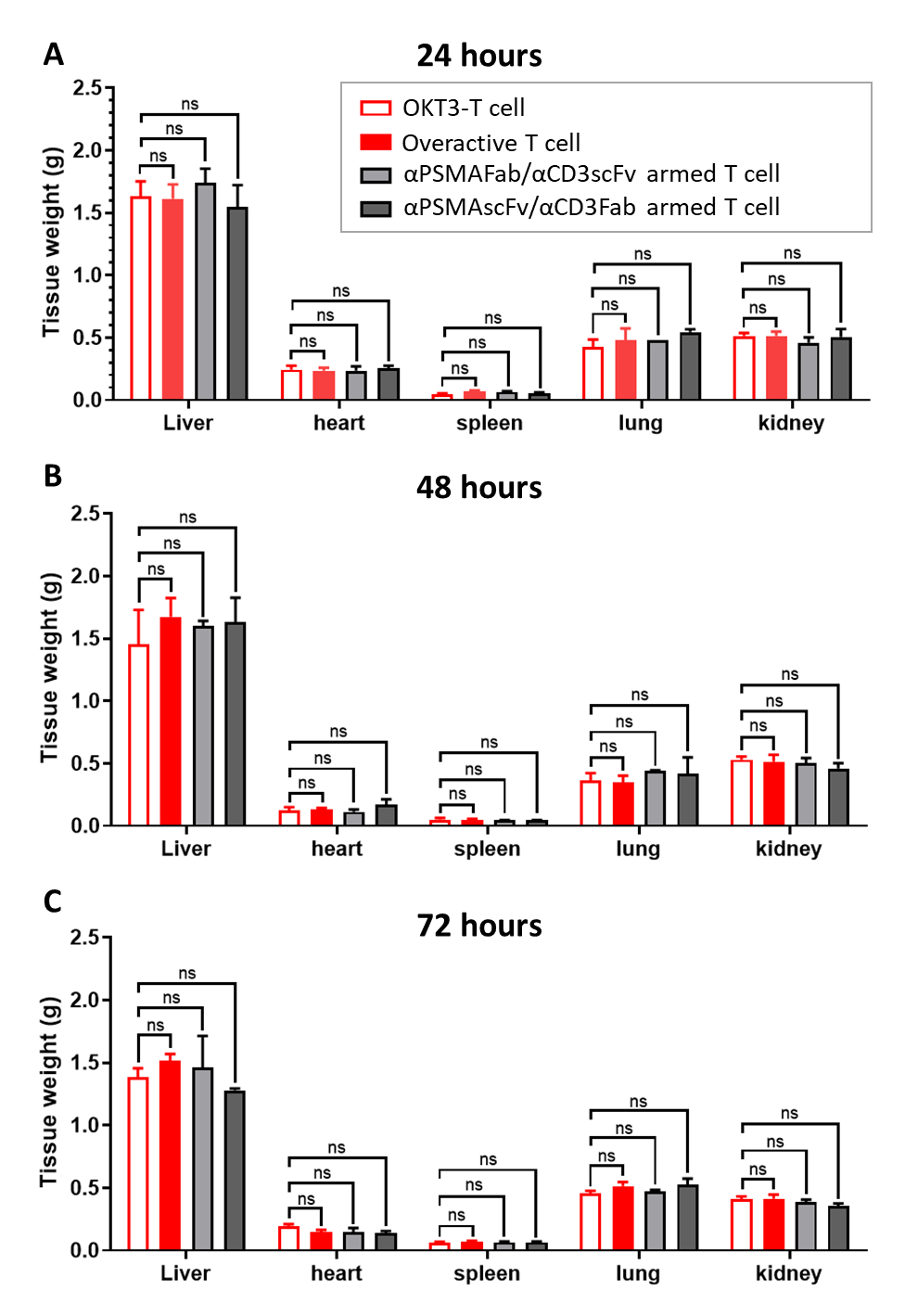
**

**Supplemental Figure 10.** **The organ weight of anti-PSMA/anti-CD3 BsAb-armed T cells treated SCID mice.** The organs were harvested from SCID mice received T cells, BsAb-armed T cells, or OKT3-induced overactivated T cells and were weighted at consecutive time points. Bar, SD. ns, not-significance.

**
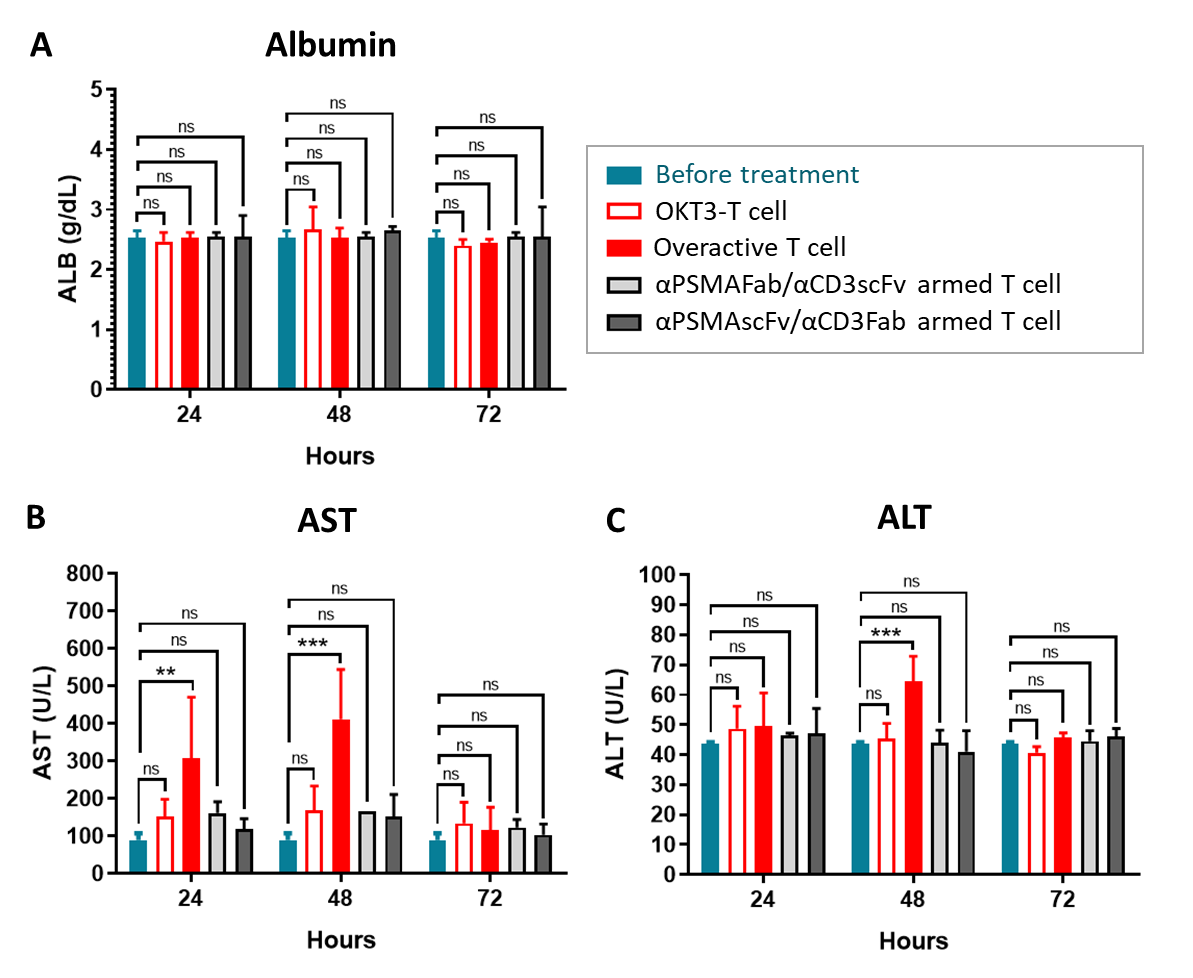
**

**Supplemental Figure 11. The liver index of T cells** **or BsAb-armed T cells treated SCID mice.** (**A**) Albumin, (**B**) AST, and (**C**) ALT in the serum of SCID mice received T cells, BsAb-armed T cells, or OKT3-induced overactivated T cells were analyzed at consecutive time points. Bar, SD. ns, not-significance. **, P<0.01. ***, P<0.001.


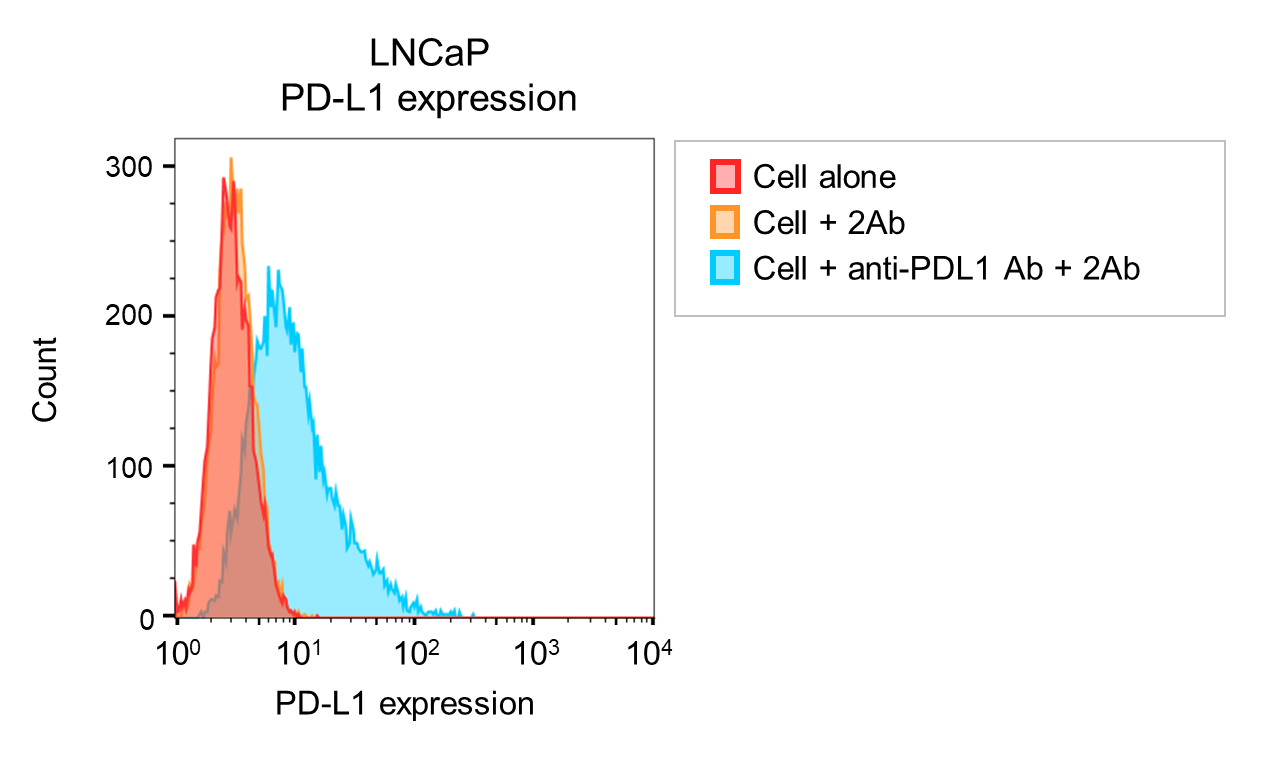


**Supplemental Figure 12.** **PD-L1 expression levels in LNCaP cell-line.** The LNCaP cells were suspended at 3×10^5^ cells/tube in PBS buffer containing 0.05% (w/v) BSA, incubated with an anti-PDL1 antibody (1 μg/mL), and detected with a FITC-conjugated goat anti-mouse IgG Fc antibody (Jackson ImmunoResearch, 1 μg/mL). The surface fluorescence of viable cells was measured using a flow cytometer (BD Biosciences, San Jose, CA, U.S.A.), and fluorescence intensities were analyzed with the Flowjo software (Treestar Inc., Ashland, OR, USA).

**
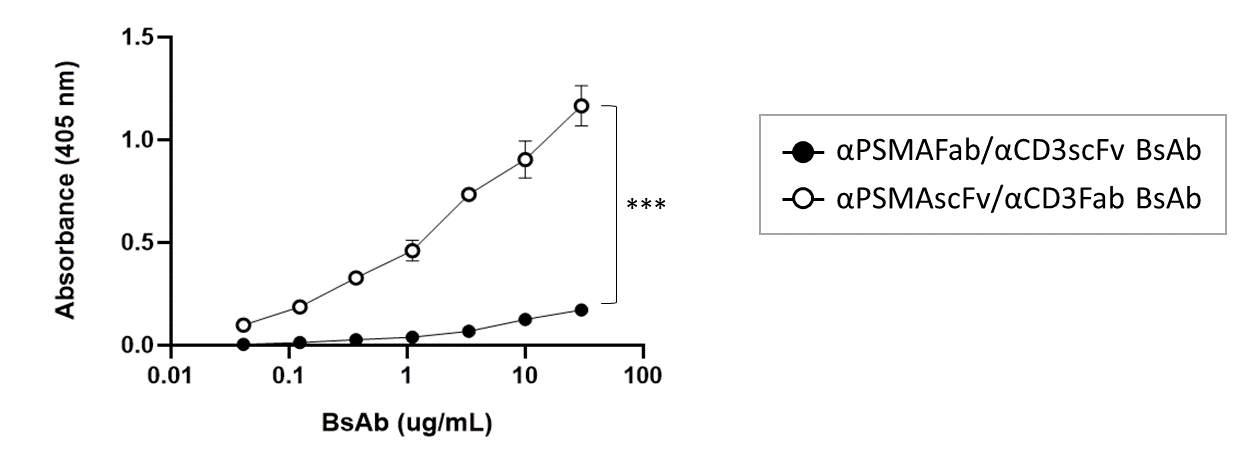
**

**Supplemental Figure 13. The human CD3 binding affinity of anti-PSMA/anti-CD3 BsAbs.** Graded concentrations (0.04, 0.12, 0.37, 1.11, 3.33, 10, and 30 μg/mL) of two types of anti-PSMA/anti-CD3 BsAbs were added into microplates coated with human CD3 molecules, and were detected by HRP-conjugated goat anti-human IgG Fab antibody. The color was developed using ABTS substrate. Bar, SD. ***, p < 0.001.

**SupplementaL Tables**

**Supplementa1 Table 1.** Maximum dose of anti-PSMA/anti-CD3 (scFv-Fab) BsAb on T cell surface.

| Maximum dose of anti-PSMA/anti-CD3 (scFv-Fab) BsAb on T cell surface (ng/10^9^ cells) | | | |
| --- | --- | --- | --- |
| Test 1 | Test 2 | Test 3 | Average ± SD |
| 34110 | 39544 | 30615 | 34756 ± 4499 |

**Supplementa1 Table 2.** The cancer killing efficiency of anti-PSMA/anti-CD3 (Fab-scFv) BsAb-armed T cells arming with different level BsAb.

|  | BsAb modified level (%) | anti-PSMA/anti-CD3 (Fab-scFv) BsAb-armed T cell | | | |
| --- | --- | --- | --- | --- | --- |
|  |  | 0% | 6.1% | 27.9% | 100% |
| Cancer killing efficiency | E:T ratio = 3:1 | 10.94±1.77 | 57.50±3.83 | 73.40±7.82 | 77.25±3.94 |
|  | E:T ratio = 5:1 | 28.04±4.68 | 77.41±4.29 | 92.29±4.37 | 94.94±2.62 |
|  | E:T ratio = 10:1 | 32.16±8.42 | 99.48±6.23 | 102.39±6.73 | 102.20±4.12 |

**Supplementa1 Table 3.** The cancer killing efficiency of anti-PSMA/anti-CD3 (scFv-Fab) BsAb-armed T cells arming with different level BsAb.

|  | BsAb modified level (%) | anti-PSMA/anti-CD3 (scFv-Fab) BsAb-armed T cell | | | |
| --- | --- | --- | --- | --- | --- |
|  |  | 0% | 17.3% | 57% | 100% |
| Cancer killing efficiency | E:T ratio = 3:1 | 10.94±1.77 | 46.99±0.66 | 59.13±6.22 | 60.79±7.38 |
|  | E:T ratio = 5:1 | 28.04±4.68 | 71.74±4.06 | 84.09±2.66 | 89.33±4.82 |
|  | E:T ratio = 10:1 | 32.16±8.42 | 100.07±6.78 | 104.88±7.72 | 98.85±3.57 |

**Supplementa1 Table 4.** The residual amount of anti-PSMA/anti-CD3 BsAbs on the surface of T cells.

|  | T cell | anti-PSMA/anti-CD3 (Fab-scFv)  BsAb-armed T cell | anti-PSMA/anti-CD3 (scFv-Fab)  BsAb-armed T cell |
| --- | --- | --- | --- |
| 0 hr | 0.00±0.00 | 100±0.00 | 100±0.00 |
| 24 hr | 0.00±0.00 | 80.94±0.79 | 94.51±20.42 |
| 48 hr | 0.00±0.00 | 67.83±32.62 | 81.81±4.93 |
| 72 hr | 0.00±0.00 | 59.55±11.49 | 72.67±4.24 |
| 96 hr | 0.00±0.00 | 39.08±10.50 | 62.88±4.09 |

**SupplementaL Movie**

**Supplemental Movie 1** (supplement to figure 3D): Time-lapse video created using live-cell images of T cells co-cultured with LNCaP.

**Supplemental Movie 2** (supplement to Figure 3D): Time-lapse video created using live-cell images of anti-PSMA/anti-CD3 (Fab-scFv) armed T cells co-cultured with LNCaP.

**Supplemental Movie 3** (supplement to Figure 3D): Time-lapse video created using live-cell images of anti-PSMA/anti-CD3 (scFv-Fab) armed T cells co-cultured with LNCaP.

**Supplemental Movie 4** (supplement to Figure 3D): Time-lapse video created using live-cell images of anti-PSMA/anti-CD3 (scFv-scFv) armed T cells co-cultured with LNCaP.

**Supplemental Movie 5** (supplement to Figure 3D): Time-lapse video created using live-cell images of anti-PSMA/anti-CD3 (hole-knob) armed T cells co-cultured with LNCaP.

**Supplemental Movie 6** (supplement to supplemental figure 6): Time-lapse video created using live-cell images of T cells co-cultured with PC-3.

**Supplemental Movie 7** (supplement to supplemental figure 6): Time-lapse video created using live-cell images of anti-PSMA/anti-CD3 (Fab-scFv) armed T cells co-cultured with PC-3.

**Supplemental Movie 8** (supplement to supplemental figure 6): Time-lapse video created using live-cell images of anti-PSMA/anti-CD3 (scFv-Fab) armed T cells co-cultured with PC-3.

**Supplemental Movie 9** (supplement to supplemental figure 6): Time-lapse video created using live-cell images of anti-PSMA/anti-CD3 (scFv-scFv) armed T cells co-cultured with PC-3.

**Supplemental Movie 10** (supplement to supplemental figure 6): Time-lapse video created using live-cell images of anti-PSMA/anti-CD3 (hole-knob) armed T cells co-cultured with PC-3.
